# Pyroptosis-Related Gene to Construct Prognostic Signature and Explore Immune Microenvironment and Immunotherapy Biomarkers in Bladder Cancer

**DOI:** 10.3389/fgene.2022.801665

**Published:** 2022-07-01

**Authors:** Xiangyu Zhang, Hengzhang Liang, Qi Tang, Hongyi Chen, Fangzhou Guo

**Affiliations:** ^1^ Department of Gastrointestinal Surgery, Guangxi Medical University Cancer Hospital, Nanning, China; ^2^ Department of Ultrasound, The First Affiliated Hospital of Guangxi Medical University, Nanning, China; ^3^ Basic Medical College, Guangxi Medical University, Nanning, China; ^4^ Department of Neurosurgery, Guangxi Medical University Cancer Hospital, Nanning, China

**Keywords:** bladder cancer, pyroptosis, overall survival, prognostic model, immunotherapy biomarkers

## Abstract

Bladder cancer is known to be the most common malignant tumor in the urinary system and has a poor prognosis; thus, new targets for drug treatment are urgently needed. Pyroptosis is defined as programmed cell death in the inflammatory form mediated by the gasdermin protein. It has therapeutic potential due to the synergistic effect of radiotherapy and chemotherapy, can reverse chemotherapy resistance, is able to regulate the body environment to alter tumor metabolism, and may enhance the response rate of the immune checkpoint inhibitor. Accordingly, this study attempted to explore the role of pyroptosis in bladder cancer. A prognostic model based on five pyroptosis-related genes was constructed by conducting univariate Cox survival and LASSO regression analyses using The Cancer Genome Atlas (TCGA) cohort. Patients were divided into high- and low-risk groups according to the median risk score, with all five PRGs having downregulated expression in the high-risk group. The high-risk group was shown to have a worse prognosis than the low-risk group, and survival differences between the two groups were then validated in the Gene Expression Omnibus (GEO) cohort. Moreover, the ROC curves demonstrated the model’s moderate predictive ability. The univariate and multivariate Cox regression analyses indicated that risk scores were found to serve as an independent prognosis factor for OS in bladder cancer patients. In addition, the high-risk group was observed to be associated with advanced N and TNM stages. A nomogram combining risk scores and clinical features was then established, with the ROC curve indicating that the AUC of TCGA training cohort in 3 and 5 years was 0.789 and 0.775, respectively. The calibration curve exhibited a high consistency between the actual survival rate and the predicted rate. Furthermore, the GO and KEGG analyses found that antigen processing and presentation of exogenous antigen, exogenous peptide antigen, and peptide antigen were enriched in the low-risk group. A higher abundance of tumor-infiltrating immune cells and additional active immune pathways were also noted in the low-risk group. In addition, immunotherapy biomarkers, including TMB, PD1, PD-L1, CTLA4, and LAG3, were shown to have higher levels in the low-risk group. Therefore, patients in the low-risk group may be potential responders to immune checkpoint inhibitors.

## Introduction

Bladder cancer (BC) is the fourth most common cancer in men and the eleventh most common in women, which has an estimated 550,000 new cases each year. In 2018, a total of 200,000 patients around the world died of UBC (Torre et al., 2015; Ferlay, 2018; Richters et al., 2020). Bladder cancer consists of non-muscle-invasive bladder cancer and muscle-invasive bladder cancer. Non-muscle invasive bladder cancer accounts for about 70% of the cases of bladder cancer (Kirkali et al., 2005), of which the mainstay treatments are based on transurethral resection of bladder tumor (TURBT) and intravesical therapy with chemotherapy or Bacille Calmette–Guérin. Muscle-invasive bladder cancer accounts for about 30% of such cases, with treatment comprising platinum-based neoadjuvant therapy followed by radical cystectomy and pelvic lymph node dissection (Ghandour et al., 2019). Only 5% of patients are initially diagnosed with metastatic bladder cancer, for which the primary treatment is cisplatin-based cytotoxic chemotherapy. Novel forms of treatments, such as targeted therapy and immunotherapy, are also widely used (Facchini et al., 2020). Despite the remarkable progress made in surgery and improvements in chemotherapy and radiotherapy, as well as the emergence of new treatment modalities, such as targeted therapy and immunotherapy, the prognosis of patients with bladder cancer remains far from satisfactory. In regard to non-muscle-invasive bladder cancer, 40–80% of patients suffer from postoperative recurrence within 1 year, with 10–25% of patients progressing to muscle-invasive bladder cancer (Dyrskjøt and Ingersoll, 2018). The five-year survival rates for muscle-invasive bladder cancer and metastatic bladder cancer are 36–48% and 5–36%, respectively (Lenis et al., 2020). Therefore, developing new drug targets to improve treatment or synergistically drive existing therapeutic measures to improve the prognosis of bladder cancer is of notable clinical significance. At the same time, establishing a prognostic gene signature model of bladder cancer in order to better predict the prognosis is urgently required.

Pyroptosis is defined as programmed cell death in the inflammatory form mediated by the gasdermin (GSDMD) protein. In contrast to other types of cell death, the associated swelling and rupture of cells release a large number of inflammatory factors and activate the immune system (Bergsbaken et al., 2009). Early studies have shown that pyroptosis cells and their related proteins play an essential role against infection (Aachoui et al., 2013; Jorgensen et al., 2017). Currently, an increasing number of studies have suggested that pyroptosis may have a dual effect on tumorigenesis and progression. Specifically, pyroptosis cells can induce the release of inflammatory cytokines and modulate the inflammatory microenvironment, thereby promoting tumor occurrence; however, pyroptosis itself can lead to the death of tumor cells and exert anti-tumor activity by influencing the EMT process, regulating the tumor microenvironment, and influencing chemotherapy resistance (Wei et al., 2014; Dupaul-Chicoine et al., 2015; Lu et al., 2018; Chen et al., 2020). In terms of the treatment of malignant tumors, pyroptosis-inducing drugs may confer synergy with radiotherapy and chemotherapy, reverse chemotherapy resistance, regulate the body environment to alter tumor metabolism, and enhance the response rate of the immune checkpoint inhibitor (Han et al., 2020; Tang et al., 2020; Zhu and Li, 2021). Therefore, developing drugs that target pyroptosis may bring about novel treatment modalities for cancer.

The risk score constructed by pyroptosis-related genes (PRGs) has been shown to be effective in predicting the prognosis, immune microenvironment, and immunotherapy response in ovarian cancer, gastric cancer, lung cancer, and glioma (Li et al., 2021; Lin et al., 2021; Shao et al., 2021; Ye et al., 2021). However, studies on PRGs in bladder cancer are lacking. Therefore, this study aims to compare the expression levels of PRGs between normal bladder and bladder cancer, build a risk score for bladder cancer, determine its prognostic and clinical values, and explore its relationship with the immune microenvironment and immunotherapy biomarkers.

## Materials and Methods

### Datasets

Data from RNA-sequencing (RNA-seq) and somatic mutation (VarScan2 Variant Aggregation and Masking), as well as the matched clinical characteristics used to construct the prognostic model, were downloaded from The Cancer Genome Atlas (TCGA) database (https://www.cancer.gov/about-nci/organization/ccg/research/structural-genomics/tcga). A total of 411 BC patient samples and 19 normal human bladder samples were obtained. RNA-seq and clinical data were downloaded from the GEO database (https://www.ncbi.nlm.nih. gov/geo/, ID: GSE13507, GSE31684) for external validation.

### Identification of Differentially Expressed Pyroptosis-Related Genes

Studies have already constructed a pyroptosis-related gene-based prognostic model in other cancers which were referred to in this study, after which a total of 33 pyroptosis-related genes were screened out (Li et al., 2021; Lin et al., 2021; Shao et al., 2021; Ye et al., 2021), as shown in Supplementary Table S1. For further comparison, the expression data of both datasets were normalized. The “limma” packages were then used to identify the differentially expressed genes (DEGs) between BC and normal tissues with a *p*-value < 0.05, with the DEGs forming a heatmap. A protein–protein interaction (PPI) network was then constructed for 33 PRGs using the Search Tool for the Retrieval of Interacting Genes (STRING), version 11.5 (https://string-db.org/). Interaction score = 0.9 was set as the lowest PPI network interaction score in order to find the hub genes.

### Consensus Clustering

A clustering analysis was conducted for 411 bladder cancer patients using the “ConsensusClusterPlus” package, which was repeated 1,000 times to make the stratification more stable. The number of clusters was determined by the K-means algorithm with the Euclidean distance. Patients were then divided into different pyroptosis modification patterns according to the K-value.

### Development and Validation of a Prognostic Model Based on Pyroptosis-Related Genes

Univariate Cox survival analysis was performed to evaluate the correlations between PRGs and prognosis in TCGA cohort. Here, *p* < 0.05 was set as the cut-off *p*-value, and the significant PRGs in the univariate Cox survival analysis were subsequently subjected to LASSO Cox regression analysis, with the minimum criteria deciding the penalty parameter (λ). The calculation formula of the risk score was: Risk score = Î£^5^_i_ Xi×Yi (X: coefficients; Y: gene expression level). BC patients in TCGA and GEO groups were then divided into high- and low-risk groups according to the median risk score, and Kaplan–Meier survival curves for OS were plotted using the survminer R package to compare the overall survival (OS) between the two groups. In order to ascertain the specificity and sensitivity of the risk score, time-dependent receiver operating characteristic (ROC) curves and AUC values were obtained using the survival ROC R package.

### Identifying Independent Prognostic Factors for OS

The clinical data (age, sex, grade, T, N, and M) as well as the risk score of each patient in both TCGA and GEO cohorts were extracted. All indicators then underwent univariate Cox survival analysis, after which the statistically significant indicators (*p* < 0.05) were incorporated into the multivariate Cox survival analysis. These indicators in the multivariate Cox survival analysis (*p* < 0.05) were considered to be independent prognostic factors.

### Developing a Prognostic Nomogram Integrated With Clinical Features and Risk Score

A nomogram was constructed based on the results of the multivariate Cox survival analysis. The prediction accuracy and discriminating ability of the nomogram were then evaluated using Harrell’s C-index and calibration curves, respectively. Furthermore, the time-dependent receiver operating characteristic (ROC) curve further evaluated the predictive performance.

### Functional Enrichment Analysis, Tumor-Infiltrating Immune Cells, and Immune-Related Pathways Between the Low- and High-Risk Groups

Using the “limma” packages, |log2FC|>log2(1.5) and FDR< 0.05 were used as the screening standard for DEGs between the high- and low-risk groups. According to these DEGs, the Gene Ontology (GO) enrichment and Kyoto Encyclopedia of Genes and Genomes (KEGG) pathway analyses were performed by applying the “clusterProfiler” package. The single sample gene set enrichment analysis (ssGSEA) was then performed to evaluate the enrichment scores of 16 tumor-infiltrating immune cells and 13 immune-related pathways in each sample from TCGA group using the R package “gsva.”

### Tumor Mutation Burden and Immune Checkpoint Analysis

The somatic mutation data (VarScan2 Variant Aggregation and Masking) were obtained from The Cancer Genome Atlas (TCGA) database. Perl scripts based on JAVA8 were used to calculate the tumor mutational burden (TMB) of each patient, after which the median TMB was set as the cut-off value in order to divide the patients into high- and low-TMB groups. The Mann–Whitney test was used to compare the difference of tumor mutation burden (TMB) and immune checkpoint genes (*PD1, PD-L1, CTLA4, LAG3, and TIM3*) between the high and low-risk groups.

## Results

### Identifying PRGs That are Differentially Expressed in Bladder Cancer and Normal Tissues

**FIGURE 1:**
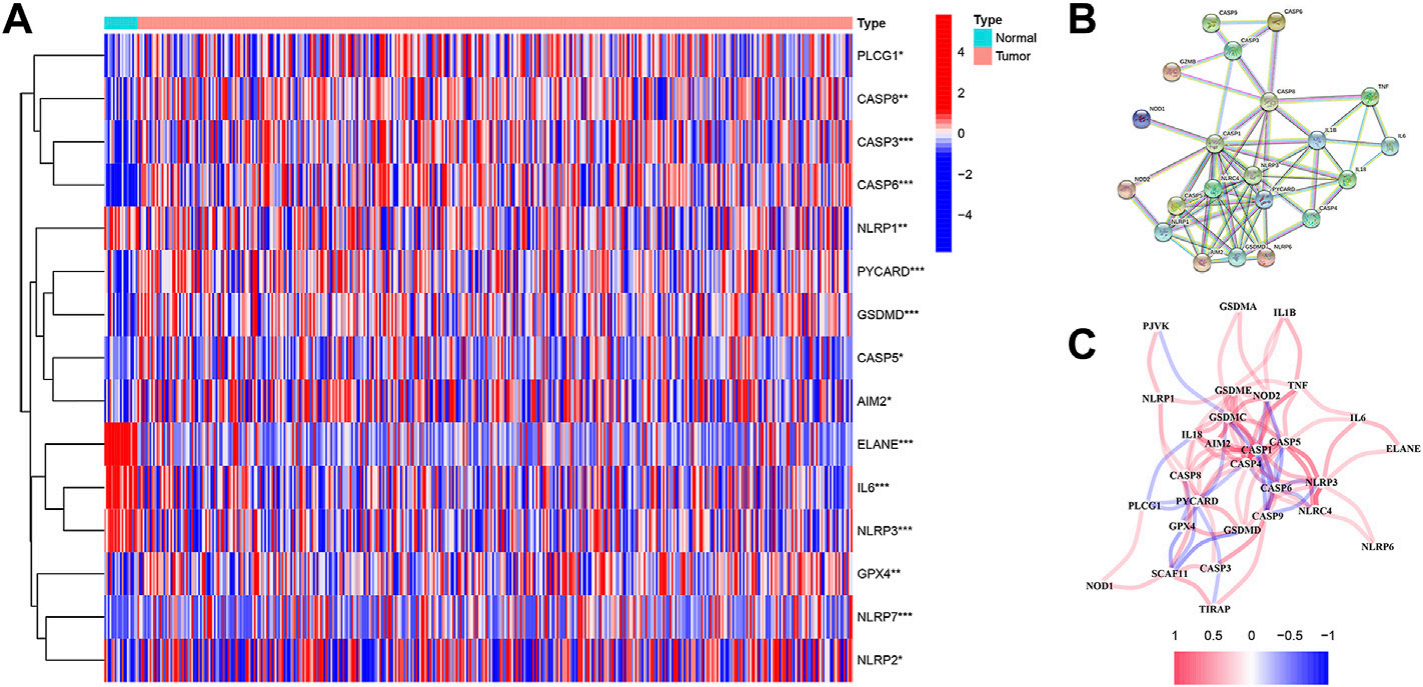
**(A)** Heatmap shows the expression levels of 15 differentially expressed PRGs between normal and tumor samples. **(B)** Protein–protein interaction (PPI) network for PRGs was constructed using the Search Tool for the Retrieval of Intervening Genes (STRING). The PPI network shows the interaction of 21 hub genes in PRGs. **(C)** Correlation network contains all 33 PRGs.

RNA sequencing (RNA-seq) data, along with the corresponding clinical data of 19 normal samples and 411 bladder cancer samples, were downloaded from TCGA database. A heatmap was constructed in order to show the differentially expressed PRGs between the normal and tumor samples (Figure 1A), of which a total of 15 PRGs were found to be differentially expressed in normal and tumor samples, while 11 genes (*AIM2, GPX4, NLRP7, NLRP2, CASP3, CASP5, CASP6, CASP8, PYCARD, PLCG1, and GSDMD*) were upregulated and four genes (*IL6, NLRP3, ELANE, and NLRP1*) were downregulated in the BC samples. In order to explore the relationship among those PRGs, a protein–protein interaction (PPI) network was implemented, with 0.9 set as the lowest PPI network interaction score. Here, 21 genes (*CASP6, CASP9, CASP3, CASP8, CASP1, CASP4, CASP5, GZMB, TNF, IL6, IL1B, IL18, NOD1, NOD2, NLRP1, AIM2, GSDMD, NLRP6, NLRC4, NLRP3, and PYCARD*) were considered hub genes (Figure 1B). Among them, 10 genes (*AIM2, CASP3, CASP5, CASP6, CASP8, GSDMD, PLCG1, PYCARD, IL6, and NLRP1*) were both hub genes and differently expressed PRGs. The results of the correlation network with all PRGs are shown in Figure 1C.

### Consensus Clustering of Bladder Cancer Based on PRGs

**FIGURE 2:**
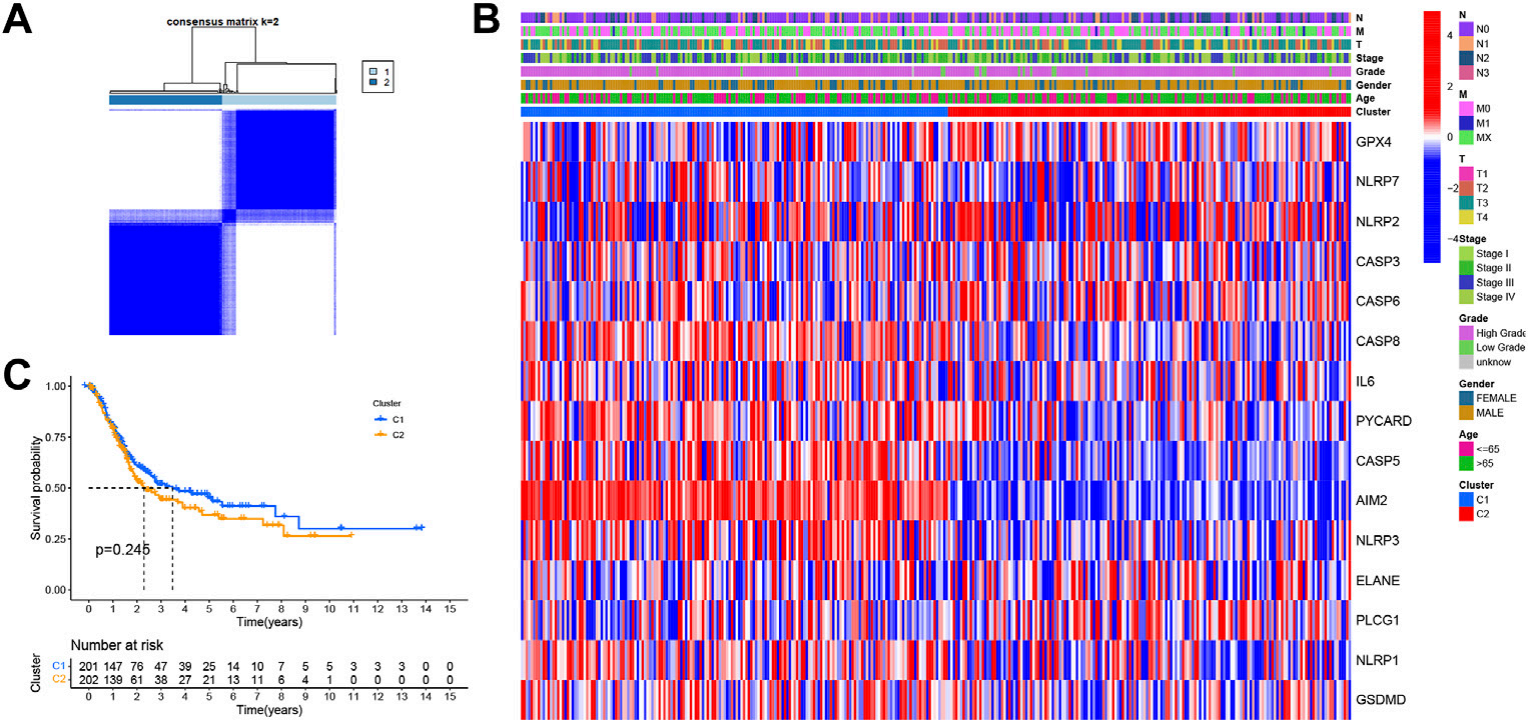
**(A)** Consensus score matrix of bladder cancer samples when K = 2. **(B)** Heatmap shows the clinical characteristics between the two subtypes, including age, sex, grade, and T, N, and M stages. **(C)** Kaplan–Meier survival curves for OS in C1 and C2 subtypes.

In order to explore the relationship between PRGs and bladder cancer subtypes, an unsupervised consensus clustering analysis was conducted for 411 bladder cancer patients downloaded from TCGA database. Accordingly, clustering variables k were found to increase from 2 to 10; when k = 2, the highest intragroup correlation and lowest intergroup correlation were obtained (Figure 2A). Therefore, patients were divided into two subtypes. The clinical features between the two subtypes are given in Figure 2B, where no significant differences were noted in regard to age, gender, T stage, N stage, M stage, and grade between the two subtypes. Kaplan–Meier survival curves of OS were then performed in order to compare the difference in prognosis between the two subtypes. The three-year survival rates of C1 and C2 were found to be 50.5 and 44.3%, respectively, while the five-year survival rates of C1 and C2 were 43.1 and 34.8%, respectively. However, the survival curves showed no significant difference in OS between C1 and C2 (*p* = 0.254) (Figure 2C).

### Extracting Prognosis Associated-PRGs and Establishing Risk Scores in TCGA Datasets

**FIGURE 3:**
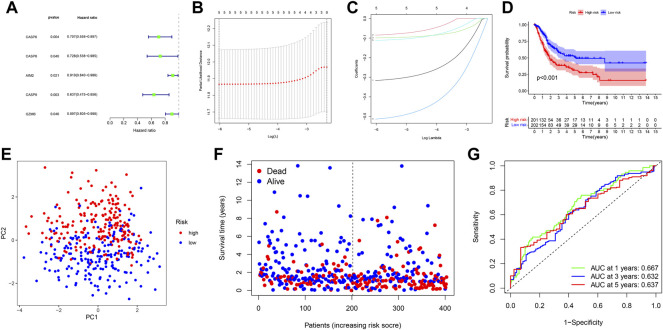
**(A)** Forest plot shows that five PRGs were potential prognostic factors for OS in univariate Cox survival analysis. **(B)** LASSO regression analysis of the five PRGs. **(C)** LASSO regression analysis of the five PRGs. **(D)** Kaplan–Meier curves for OS in bladder cancer patients (*n* = 403) who had been stratified into high- and low-risk groups based on the median risk score. **(E)** PCA plot shows the distribution of high-risk and low-risk groups. **(F)** Distribution of the survival status for the high-risk and low-risk groups. **(G)** ROC curves of 1, 3, and 5 years for the risk score.

In order to further assess the prognostic value of PRGs, PRG-based risk scores were constructed to predict bladder cancer patient survival. Here, 403 patients with complete survival data in the TCGA database underwent univariate Cox survival analysis for 33 PRGs, of which five PRGs were found to be statistically significant (Figure 3A). A LASSO regression analysis was then carried out in order to construct the prognostic model, with a penalty parameter (λ) of 5 (Figure 3B,C). The formula used to calculate the risk score was: (−0.3168) * CASP6+ (-0.0849) * CASP8+ (−0.0992) *AIM2+ (−0.5139) * CASP9+ (−0.1118) * GZMB. All patients were separated into the high-risk group (*n* = 201, risk score ≥ −4.972744) and low-risk group (*n* = 202, risk score < −4.972744) based on the median cut-off value. The Kaplan–Meier survival curve for OS showed that the high-risk group had a shorter survival time than the low-risk group (*p* < 0.001) (Figure 3D). Moreover, the principal component analysis (PCA) showed that patients in different risk groups were distributed into two different groups (Figure 3E). Specifically, patients in the high-risk group had a higher possibility of death and a shorter survival time than those in the low-risk group (Figure 3F). A time-dependent ROC curve was then created in order to evaluate the performance of the prediction risk score. Accordingly, the AUC values of the risk score in 1, 3, and 5 years were 0.667, 0.632, and 0.637, respectively (Figure 3G).

### Validating the Predictive Efficiency of the Risk Model in the GEO Cohort

**FIGURE 4:**
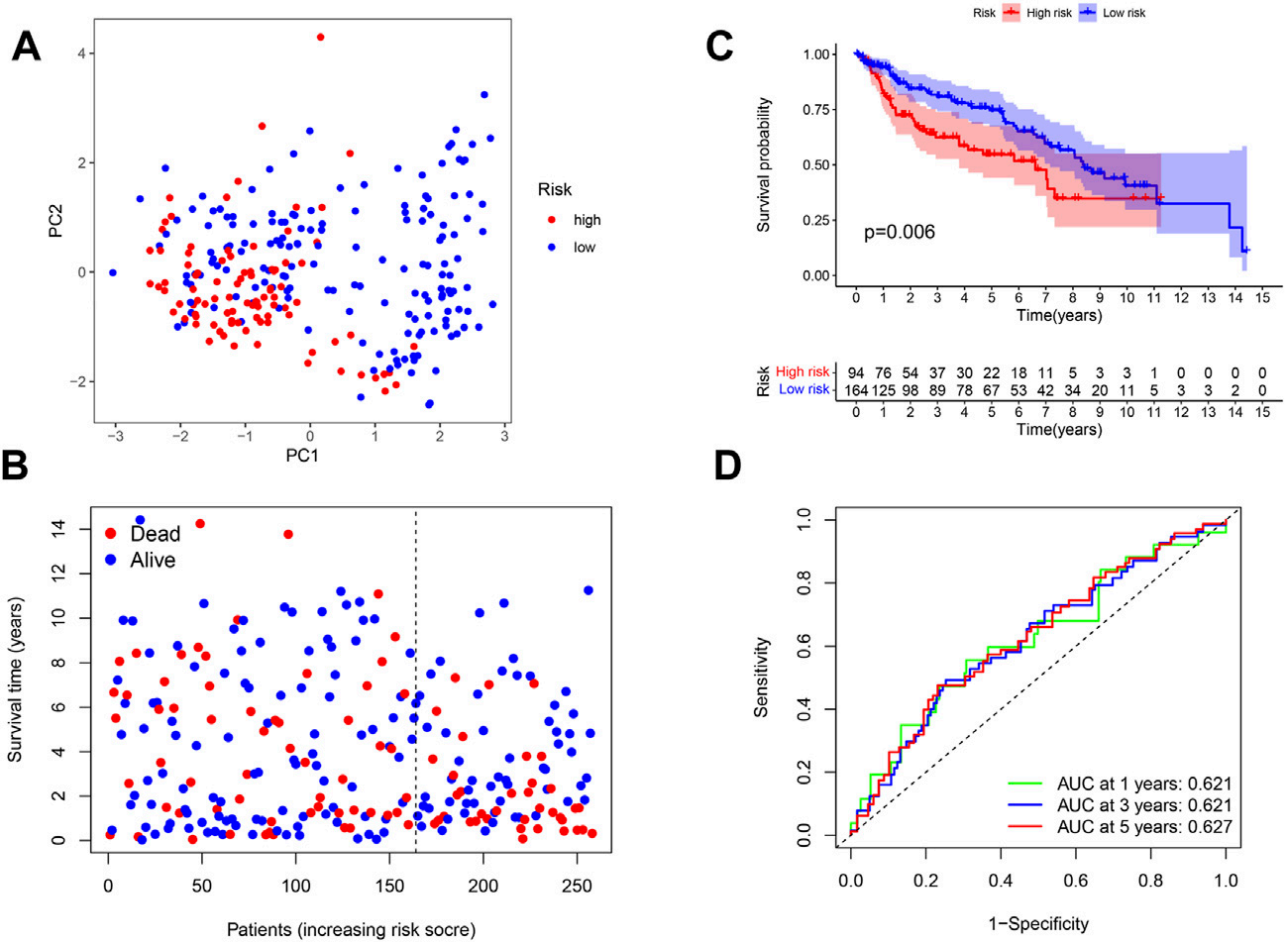
**(A)** PCA plot shows the distribution of high-risk and low-risk groups. **(B)** Distribution of the survival status for the high-risk and low-risk groups. **(C)** Kaplan–Meier curves of OS for the high-risk and low-risk groups (*p* = 0.006). **(D)** ROC curves of 1, 3, and 5 years for the risk score.

In order to validate the replicability of the risk score in another patient group, PRG expression in 258 patients with complete survival data was obtained from the GEO cohort (GSE13507 and GSE31684). The GEO dataset was then divided into the high-risk group (*n* = 94) and low-risk group (*n* = 164) based on the median risk score cut-off. PCA showed that patients in the two risk groups were also distributed according to two different groups (Figure 4A). As shown in Figure 4B, the high-risk group was noted to have more death events, while the low-risk group had a higher probability of survival (Figure 4B). As illustrated by the Kaplan–Meier curves for OS in Figure 4C, patients in the high-risk group were found to have a worse prognosis than those in the low-risk group (*p* = 0.006), with the high-risk group having a shorter survival time. To further assess the accuracy of the predictive risk model, a time-dependent ROC curve was analyzed. Here, the AUC values of the model in 1, 3, and 5 years were found to be 0.621, 0.621, and 0.627, respectively (Figure 4D).

### Risk Score is an Independent Prognosis Factor for OS.

**FIGURE 5:**
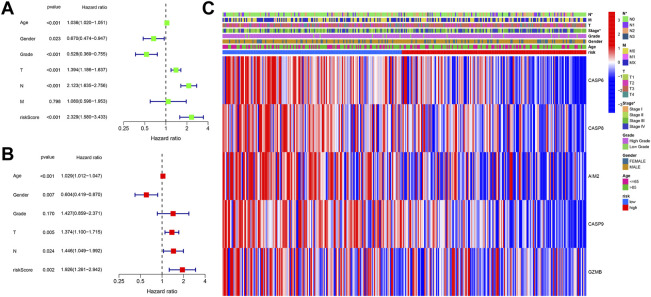
**(A)** Univariate Cox regression analysis of OS for clinical characteristics and risk score. **(B)** Multivariate Cox regression analysis of clinical characteristics and risk score. **(C)** Heatmap shows the connections between clinical characteristics and risk score.

Patients were integrated from TCGA and GEO datasets as a single dataset group to explore factors influencing the prognosis. The clinical characteristics (age, sex, grade, T, N, and M) and risk score in the two datasets were then analyzed *via* univariate and multivariate Cox analyses. In the univariate Cox analysis, variables including T, N, age, gender, and risk score were found to serve as significant prognostic factors for prognosis (Figure 5A). Moreover, risk score, gender, T, and N were verified as independent prognostic factors for OS according to the multivariate Cox regression analysis (Figure 5B). In order to explore the relationship between risk score and clinical features, a heatmap was constructed. As shown in Figure 5C, patients in the high-risk group were observed to be associated with advanced N and TNM stages.

### A Nomogram Integrated with Risk Scores and Clinical Signatures

**FIGURE 6:**
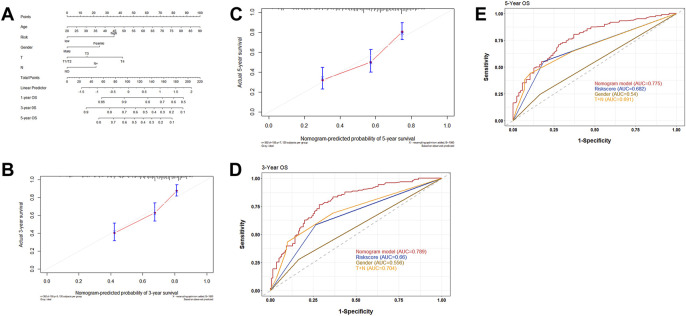
**(A)** Nomograms integrated with clinical characteristics and risk score for predicting OS based on all the patients in two cohorts. **(B)** Calibration curves predicting 3-year OS. **(C)** Calibration curves predicting 5-year OS. **(D)** ROC curve for 3 years to assess the AUC of the gender, nomogram, and T + N stage. **(E)** ROC curve for 5 years to assess the AUC of the gender, nomogram, and T + N stage.

To clarify the prognostic value of the risk score in clinical application, a nomogram integrating clinical features and risk score was established. Gender, age, T, N, and risk score were then verified as independent prognostic factors for the OS in the multivariate Cox regression analysis and were used as variables to construct the nomogram (Figure 6A). The C-index for the nomogram was found to be 0.717 (CI 95%: 0.635–0.799). The calibration curve demonstrated a high consistency between the actual survival rate and predicted survival rate in 3 and 5 years (Figure 6B,C). As seen in the ROC curve, the AUC values of the nomogram in 3 and 5 years were found to be 0.789 and 0.775, respectively, which was higher than the AUC values of gender, risk score, and T + N stage (Figure 6D,E).

### Functional Analysis for the PRG Risk Score

**FIGURE 7:**
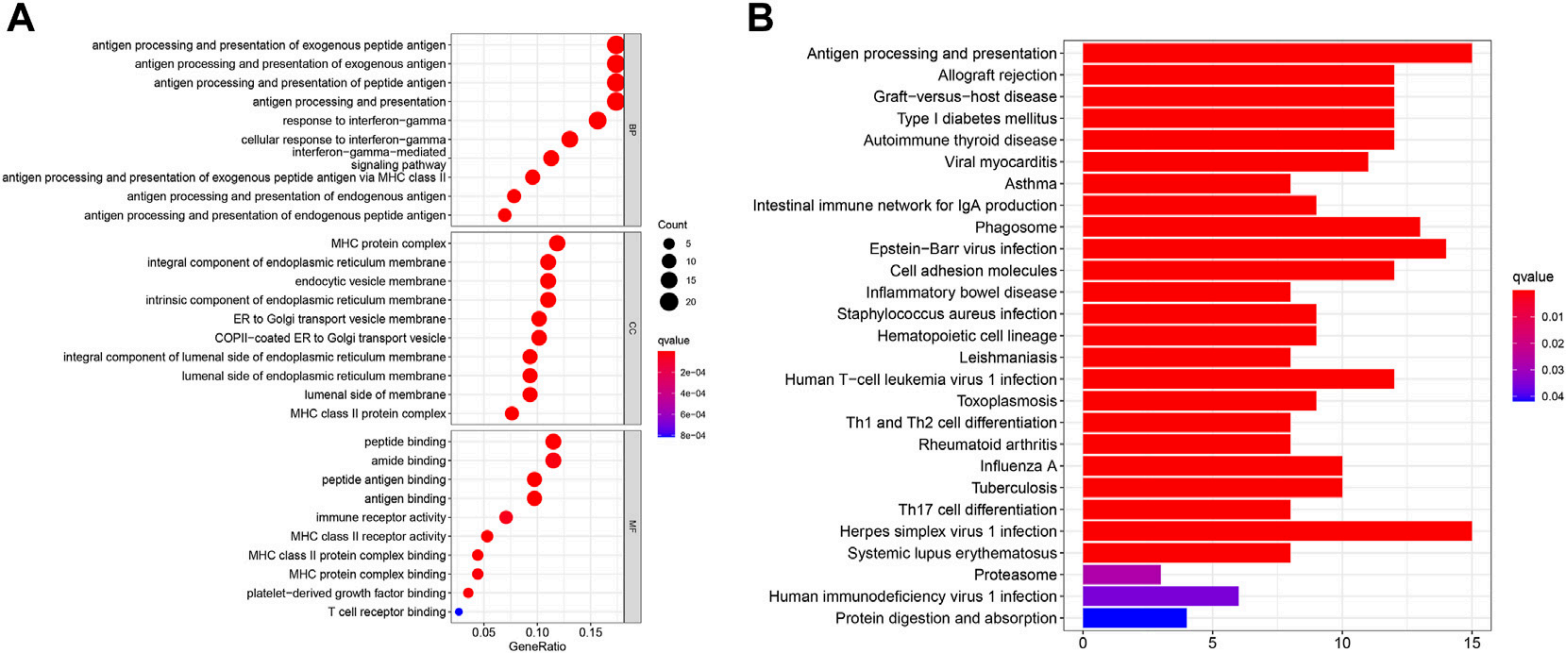
**(A)** Bubble graph shows the analysis of GO enrichment for DEGs between two risk groups. **(B)** Barplot graph shows the analysis of KEGG enrichment for DEGs between two risk groups.

GO and KEGG pathway analyses were then conducted in order to explore the potential mechanism and biological function at the gene level for both the high- and low-risk groups. The “limma” R package was used to extract the differentially expressed genes (DEGs) between the two groups, with standards FDR< 0.05 and |log2FC|>log2 (1.5). A total of 122 DEGs were screened, of which 94 genes were found to be downregulated while 28 genes were upregulated in the high-risk group. The GO analysis showed that the genes were more involved in immunity, especially in the biological process of antigen processing and presentation of exogenous peptide antigen, exogenous antigen, and peptide antigen (Figure 7A). Meanwhile, the KEGG analysis showed that these genes were also more correlated with antigen processing- and presentation-related pathways (Figure 7B).

### The Tumor-Infiltrating Immune Cells and Immune-Related Pathways Between Two Risk Groups

**FIGURE 8:**
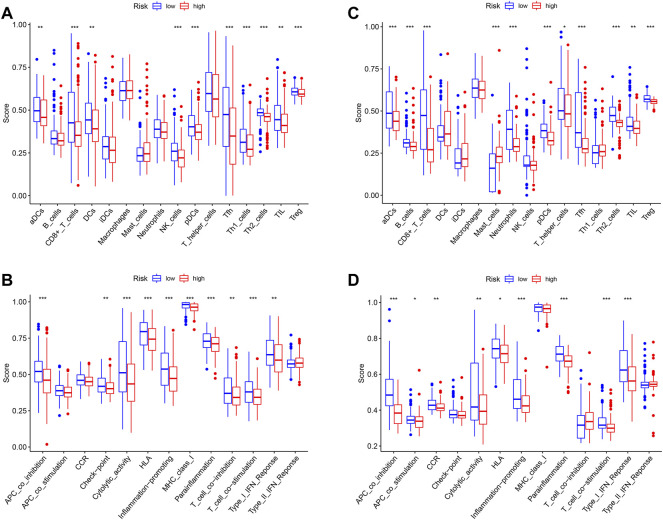
**(A)** Enrichment levels of 16 tumor-infiltrating immune cells in high- and low-risk groups from TCGA data. **(B)** Enrichment levels of 13 immune-related pathways in high- and low-risk groups from TCGA data. **(C)** Enrichment levels of 16 tumor-infiltrating immune cells in high- and low-risk groups from GEO data. **(D)** Enrichment levels of 13 immune-related pathways in high- and low-risk groups from GEO data.

In order to explore differences in the tumor microenvironment (TME) between the high- and low-risk groups, a single-sample gene set enrichment analysis (ssGSEA) was conducted to evaluate the enrichment scores of 16 tumor-infiltrating immune cells and 13 immune-related pathways in each sample from TCGA and GEO groups. The enrichment scores of aDCs, CD8+_T_cells, pDCs, Tfh, Th2_cells, TIL, and Treg in the low-risk group were found to be higher than those in the high-risk group in both TCGA and GEO cohorts (Figure 8A,C). The enrichment level of DCs, NK_cells, and Th1_cells was noted to be higher in the low-risk group in TCGA cohort, while the enrichment level of B_cells, mast_cells, neutrophils, and T_helper_cells was observed to be higher in the low-risk group in the GEO cohort. Figures 8B,D show that seven immune-related pathways were found to be more significantly enriched in the low-risk group of both TCGA and GEO cohorts, including APC_co_inhibition, cytolytic_activity, HLA, inflammation promoting, parainflammation, T_cell_co-stimulation, and Type_I_IFN_Reponse. The immune-related pathways of checkpoints, MHC_class_I and T_cell_co-inhibition, were shown to be higher in the low-risk group of TCGA cohort, while APC_co_stimulation and CCR were higher in the low-risk group of the GEO cohort. These results suggest that the low-risk group had a higher abundance of tumor-infiltrating immune cells as well as a more active immune pathway than the high-risk group, which may explain the difference in prognosis between the two groups.

### The Difference in the Level of Immunotherapy Biomarkers Between the Two Groups

**FIGURE 9:**
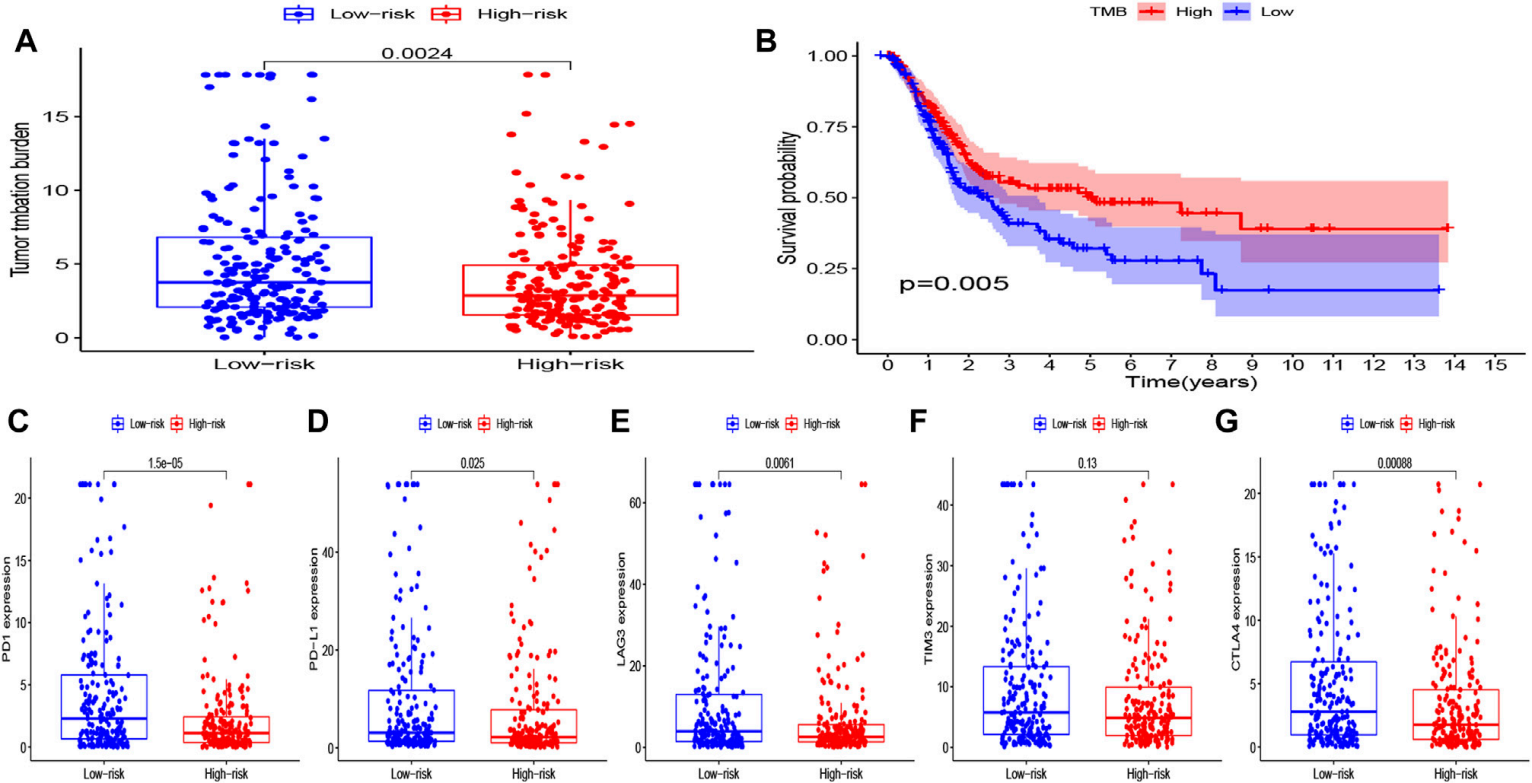
**(A)** Level of TMB in the high- and low-risk groups. **(B)** Kaplan–Meier curves of OS for the high- and low-TMB groups (P = 0.005). **(C–G)** Level of immune checkpoints (PD1, PD-L1, TIM3, LAG3, and CTLA4) in the high- and low-risk groups.

In exploring the potential effect of immunotherapy in both groups, the difference of immunotherapy biomarkers was investigated between the two groups. Accordingly, heterogeneity in the tumor mutation burden was noted between the two groups, with the low-risk group having a higher TMB than the high-risk group (Figure 9A). The prognostic value of TMB in bladder cancer was also examined, in which the Kaplan–Meier survival curve showed that the high-TMB group had a significantly longer OS than that of the low-TMB group (*p* = 0.005) (Figure 9B). In addition, the levels of other immunotherapy biomarkers were investigated (PD1, PD-L1, CTLA4, LAG3, and TIM3) in the high- and low-risk groups. Here, PD1, PD-L1, CTLA4, and LAG3 were all noted to be highly expressed in the low-risk group, while TIM3 expression exhibited no difference between the two groups (Figure 9C–G). The corresponding findings suggest that patients in the low-risk group may potentially respond to immunotherapy.

## Discussion

Pyroptosis is defined as inflammatory programmed death mediated by intracellular inflammasome and the gasdermin D (GSDMD) protein, which may have a dual effect on tumorigenesis and progression. Specifically, pyroptosis can significantly influence the immune-related signal cascade and further shape the inflammatory microenvironment, which may be beneficial to tumorigenesis; however, pyroptosis itself can lead to tumor cell death and carry out anti-tumor activities through modulation of the EMT process, regulation of the tumor microenvironment, and influencing chemotherapy resistance (Wei et al., 2014; Dupaul-Chicoine et al., 2015; Lu et al., 2018; Chen et al., 2020). According to relevant studies, drugs inducing pyroptosis may have an impact on the synergism of radiotherapy and chemotherapy, reversal of chemotherapy resistance, regulation of the body environment in shaping tumor metabolism, and enhancement of the response rate of the immune checkpoint inhibitor (Han et al., 2020; Tang et al., 2020; Zhu and Li, 2021). Moreover, tumor cells have been shown to hijack the caspase-9 pathway in order to inhibit radiotherapy, which may improve radiotherapy sensitivity (Zhang et al., 2019). In melanoma, low-GSDME expression has been described to cause tumor cells to be resistant to etoposide, with the activation of pyroptosis, reversing chemotherapy drug resistance. In addition, pyroptosis-inducing drugs may enhance the efficacy of immune checkpoint inhibitors (ICIs) and transform a “cold tumor” into a “hot tumor.” ICIs have been widely used in the clinical management of malignant tumors; however, only one-third of patients respond to them (Jiang et al., 2018). The combination of ICIs and pyroptosis-inducing drugs may also make “cold tumors” respond (Tang et al., 2020). One study found that inhibition of casp-9 can induce PD-L1 upregulation in colon cancer cells, demonstrating the potential efficacy of ICIs in conjunction with pyroptosis-inducing drugs (Han et al., 2020). Wang et al. (2020) found that pyroptosis of less than 15% of tumor cells was sufficient to clear the entire 4T1 breast tumor xenograft in an animal model. Furthermore, in regard to “cold tumors” that do not respond to immune checkpoint inhibitors, the synergistic treatment of GSDM and PD1 inhibitors has been shown to trigger tumor response.

This study constructed a pyroptosis gene signature prognostic model in bladder cancer. RNA-sequencing (RNA-seq) data, along with the matched clinical characteristics of bladder cancer patients, were obtained from TCGA database, after which the risk score was established by conducting univariate Cox survival and LASSO regression analyses. Eventually, five PRGs were screened so as to establish the prognostic model, with all being favorable factors for prognosis. According to the median risk score of TCGA cohort, patients were divided into high- and low-risk groups. In TCGA cohort, the Kaplan–Meier survival curve showed that the OS of the high-risk group was significantly lower than that of the low-risk group. According to the ROC curve, the AUCs of the model at 1, 3, and 5 years were found to be 0.667, 0.632, and 0.637, respectively. In the GEO cohort, the Kaplan–Meier survival curve also showed that the OS in the high-risk group was worse than that in the low-risk group. Meanwhile, the AUCs at 1, 3, and 5 years were 0.621, 0.621, and 0.627, respectively. A nomogram combined with risk score and clinical features was then established in order to facilitate clinical use, of which the AUC values of the nomogram in 3 and 5 years were found to be 0.789 and 0.775, respectively, which were higher than the AUC values of gender, risk score, and T + N stage. According to the GO and KEGG enrichment analyses, the DEGs between the two risk groups were shown to be more involved in antigen processing and presentation. In addition, a higher abundance of tumor-infiltrating immune cells and additional active immune pathways was observed in the low-risk group. Immunotherapy biomarkers were investigated in both groups, where the low-risk group was found to be rich in TMB, PD1, PD-L1, CTLA4, and LAG3. This suggests that patients in the low-risk group may potentially be responsive to immunotherapy.

Pyroptosis plays a bidirectional role in the occurrence and development of tumors. The activation of NLRP3 has been shown to promote the secretion of α-SMA and type I collagen as well as other fibrosis markers to promote liver fibrosis, thus promoting the occurrence of hepatocellular carcinoma (Li et al., 2019). However, in hepatocellular carcinoma tissues, the expression of NLRP3 has been shown to be negatively correlated with the pathological grade and clinical stage (Wei et al., 2014), suggesting that apoptosis inhibits the further development of tumors. Similar results were evident in the present study, in which the genetic expression of three genes (AIM2, CASP6, and CASP8) was upregulated in tumor tissues and was validated as protective factors in the multivariate Cox regression analysis. Thus, these genes may play a similar role in bladder cancer as NLRP3 in liver cancer.

AIM2 is a pattern recognition receptor for the pyroptosis pathway, which is composed of the HIN-200 domain and N-terminal PYD. The HIN-200 domain binds with dsDNA, while the N-terminal PYD recruits ASC and CASP-1 to co-assemble into inflammasomes that dissociate the precursors of IL-1β and IL-18 into mature IL-1β and IL-18, thereby initiating cell pyroptosis (Yu et al., 2021). In this study, high levels of AIM2 were found to be a protective factor for bladder cancer, though its level of expression and prognostic value were shown to be contradictory across different tumors. In colon cancer, decreased expression of AIM2 is known to be associated with advanced stages of cancer and tumor progression, while low AIM2 expression is considered to be indicative of poor prognosis (Zhao et al., 2019). In gastric cancer, the upregulation of AIM2 expression has been confirmed to promote tumorigenesis in mouse models, while target blocking AIM2 can inhibit tumor occurrence. Moreover, the survival rate of patients with high AIM2 expression has been shown to be lower than those with low AIM2 expression (Dawson et al., 2021). Another study found that radiotherapy can activate the AIM2/NLRP3-CasPA-IL-1 signaling pathway in mouse models, promoting tumor elimination (Han et al., 2021). Caspase-6 is considered to mediate the activation of apoptotic caspase (Van Opdenbosch and Lamkanfi, 2019), which is a crucial regulator of innate immunity, inflammasome activation, and host defense (Foveau et al., 2014). However, the function of caspase-6 is not limited to apoptosis; it is a crucial component structure of ZBP1-PANoptosome, which can induce PANoptosis (“P,” pyroptosis; “A,” apoptosis; “N,” necroptosis; and “optosis”) to prevent IAV infection (Zheng and Kanneganti, 2020). However, few studies pertaining to caspase-6 in malignant tumors exist. This study showed that caspase-6 serves as a prognostic factor for bladder cancer, though further investigation is needed to explore the role of caspase-6 in the pyroptosis-related pathway as well as ascertain its clinical value. Caspase-8 is the molecular switch of apoptosis, necrosis, and pyroptosis (Fritsch et al., 2019), and activated Caspase-8 can directly cleave GSDMD, thereby inducing cell pyroptosis (Orning et al., 2018; Sarhan et al., 2018). Caspase-8 expression levels were found to be lower in normal brain tissue than those in malignant glioma, while patients with overexpression of caspase-8 were in the earlier stages. At the cellular level, caspase-8 can inhibit malignant glioma cell proliferation (Wang et al., 2017). Studies have shown that tumor cells suppress radiation-induced immunity by hijacking caspase-9 signaling. Moreover, inhibiting caspase-9 can induce tumor cells to produce type I interferon, increase activity of tumor-specific CD8+T cells, delay tumor cell growth, and increase radiotherapy sensitivity (Han et al., 2020). The findings of the present study suggest that targeted CASP-9 inhibitors may have the clinical potential to enhance the efficacy of radiotherapy. In triple-negative breast cancer, Mir-224 has been shown to downregulate caspase-9 expression and promote tumor growth (Zhang et al., 2019). GZMB acts as a molecular weapon for cytotoxic lymphocytes in defending against viral infection and malignant transformation (Kurschus and Jenne, 2010). GZMB rapidly cleaves CASP-3 in target cells and activates the CASP-3/GSDME-mediated pyroptosis pathway (Liu et al., 2020). A recent study has also shown that GZMB can cleave GSDME and directly induce cell pyroptosis (Zhang et al., 2020). Furthermore, in patients with stage IV non-small cell lung cancer who were treated with ICIs, high levels of GZMB resulted in better OS and PFS (Hurkmans et al., 2020). In colon tumors, GZMB has been shown to be highly expressed in the proximal colon and is characterized by MSI-high and BRAF mutations, with higher levels of GZMB being associated with longer OS and CSS (Prizment et al., 2017). The aforementioned studies all indicate the potential of utilizing pyroptosis genes in clinical settings.

Pyroptosis-related target drugs possess great potential in the development of tumor drugs. Induced pyroptosis can activate anti-tumor immunity, suggesting that the combination of immune checkpoint inhibitors and pyroptosis-inducing drugs may lead to a higher response rate. Inhibition of cell apoptosis serves as the mechanism of tumor resistance to chemotherapy and radiotherapy. Cell pyroptosis is a new form of programmed cell death, and the development of drugs targeting pyroptosis may serve as a novel approach in overcoming drug resistance. The heterogeneity and deletion of tumor antigens are one of the reasons for the failure of adoptive immunotherapy (Titov et al., 2021). Pyroptosis of tumor cells can induce the release of tumor antigens, promote the maturation of DCB cells, initiate T-cell cloning and proliferation, and activate anti-tumor immunity, which may be beneficial in order to enhance the efficacy of adoptive immunotherapy.

This study has several limitations. First, the number of normal tissue samples in TCGA cohort was relatively small, which may lead to bias. Second, only one case of non-muscle-invasive bladder cancer was present in the data; hence, the results may be limited to muscle-invasive bladder cancer. In addition, *in vivo* and *in vitro* experiments are necessary to support the findings of this study.

## Data Availability

The original contributions presented in the study are included in the article/Supplementary Material; further inquiries can be directed to the corresponding author.
