# Supplementary material for: Pyroptosis-Related Gene to Construct Prognostic Signature and Explore Immune Microenvironment and Immunotherapy Biomarkers in Bladder Cancer
Source: Front Genet. 2022 Jul 1;13:801665. doi: 10.3389/fgene.2022.801665 (PMC9283834; doi:10.3389/fgene.2022.801665)
Supplement: Supplementary file 2 [file DataSheet1.PDF]

Pyroptosis-related gene to construct prognostic signature,explore immune microenvironment and immunotherapy biomarkers in bladder cancer

Running title: Pyroptosis-related gene in bladder cancer

Xiangyu Zhang[1], Hengzhang Liang[2], Qi Tang[3], Hongyi Chen[3], Fangzhou Guo[4]*

1. Department of gastrointestinal surgery， Guangxi Medical University Cancer Hospital, Nanning , Guangxi Zhuang Autonomous Region, China;

2. Department of ultrasound，The First Affiliated Hospital of Guangxi Medical University， Nanning, Guangxi Zhuang Autonomous Region, China;

3. Basic Medical College， Guangxi Medical University， Nanning, Guangxi Zhuang Autonomous Region, China;

4. Department of Neurosurgery, Guangxi Medical University Cancer Hospital, Nanning, Guangxi Zhuang Autonomous Region, China;

Corresponding author:Fangzhou Guo* guofangzhou@gxmu.edu.cn

ABSTRACT

Bladder cancer is known to be the most common malignant tumor in the urinary system and has a poor prognosis; thus, new targets for drug treatment are urgently needed. Pyroptosis is defined as programmed cell death in the inflammatory form mediated by the Gasdermin protein. It has therapeutic potential due to the synergistic effect of radiotherapy and chemotherapy, can reverse chemotherapy resistance, is able to regulate the body environment to alter tumor metabolism, and may enhance the response rate of the immune checkpoint inhibitor. Accordingly, this study attempted to explore the role of pyroptosis in bladder cancer. A prognostic model based on 5 pyroptosis-related genes was constructed by conducting univariate Cox survival and LASSO regression analyses using The Cancer Genome Atlas (TCGA) cohort. Patients were divided into high and low-risk groups according to the median risk score, with all 5 PRGs having downregulated expression in the high-risk group. The high-risk group was shown to have a worse prognosis than the low-risk group, and survival differences between the two groups were then validated in the Gene Expression Omnibus (GEO) cohort. Moreover, the ROC curves demonstrated the model's moderate predictive ability. The univariate and multivariate

Cox regression analyses indicted risk score were found to serve as an independent prognosis factor for OS in bladder cancer patients. In addition, the high-risk group was observed to be associated with advanced N and TNM stages. A nomogram combined risk score and clinical features were then established, with the ROC curve indicating that the AUC of the TCGA training cohort in 3 and 5 years was 0.789 and 0.775, respectively. The calibration curve exhibited a high consistency between the actual survival rate and the predicted rate. Furthermore, the GO and KEGG analyses found that the antigen processing and presentation of exogenous antigen, exogenous peptide antigen, and peptide antigen were enriched in the low-risk group. A higher abundance of tumor-infiltrating immune cells and additional active immune pathways were also noted in the low-risk group. In addition, immunotherapy biomarkers, including TMB, PD1, PD-L1, CTLA4 and LAG3 were shown to have higher levels in the low-risk group. Therefore, patients in the low-risk group may be potential responders to immune checkpoint inhibitors.

Keywords: bladder cancer, pyroptosis, overall survival, a prognostic model, immunotherapy biomarkers

INTRODUCTION

Bladder cancer (BC) is the fourth most common cancer in men and the eleventh most common in women, which has an estimated 550,000 new cases each year. In 2018, a total of 200,000 patients around the world died of UBC.[1-3] Bladder cancer consists of non-muscle invasive bladder cancer and muscle-invasive bladder cancer. Non-muscle invasive bladder cancer accounts for about 70% of cases of bladder cancer [4], of which the mainstay treatments are based on transurethral resection of bladder tumor (TURBT) and intravesical therapy with chemotherapy or Bacille Calmette-Guérin. Muscle-invasive bladder cancer accounts for about 30% of such cases, with treatment comprising platinum-based neoadjuvant therapy followed by radical cystectomy and pelvic lymph node dissection.[5] Only 5% of patients are initially diagnosed with metastatic bladder cancer, for which primary treatment is cisplatin-based cytotoxic chemotherapy. Novel forms of treatment, such as targeted therapy and immunotherapy, are also widely used.[6] Despite the remarkable progress made in surgery, improvements in chemotherapy and radiotherapy, as well as the emergence of new treatment

modalities, such as targeted therapy and immunotherapy, the prognosis of patients with bladder cancer remains far from satisfactory. In regard to non-muscle invasive bladder cancer, 40% to 80% of patients suffer from postoperative recurrence within 1 year, with 10% to 25% of patients progressing to muscle-invasive bladder cancer. [7] The 5-year survival rates for muscle-invasive bladder cancer and metastatic bladder cancer are 36% to 48% and 5% to 36%, respectively.[8] Therefore, developing new drug targets to improve treatment or synergistically drive existing therapeutic measures to improve the prognosis of bladder cancer is of notable clinical significance. At the same time, establishing a prognostic gene signature model of bladder cancer in order to better predict prognosis is urgently required.

Pyroptosis is defined as programmed cell death in the inflammatory form mediated by the Gasdermin （GSDMD） protein. In contrast to other types of cell death, the associated swelling and rupture of cells release a large number of inflammatory factors and activate the immune system [9]. Early studies have shown that pyroptosis cells and their related proteins play an essential role against infection. [10-11] Currently, an increasing number of studies have suggested that pyroptosis may have a dual effect on tumorigenesis and progression. Specifically, pyroptosis cells can induce the release of inflammatory cytokines and modulate the inflammatory microenvironment, thereby promoting tumor occurrence; however, pyroptosis itself can lead to the death of tumor cells and exert anti-tumor activity by influencing the EMT process, regulating the tumor microenvironment, and influencing chemotherapy resistance.[12-15] In terms of the treatment of malignant tumors, pyroptosis-inducing drugs may confer synergy with radiotherapy and chemotherapy, reverse chemotherapy resistance, regulate the body environment to alter tumor metabolism, and the enhance the response rate of the immune checkpoint inhibitor.[16-18] Therefore, developing drugs that target pyroptosis may bring about novel treatment modalities for cancer.

The risk score constructed by pyroptosis-related genes (PRGs) has been shown to be effective in predicting the prognosis, immune microenvironment, and immunotherapy response in ovarian cancer, gastric cancer, lung cancer, and glioma [19-22]. However, studies on PRGs in bladder cancer are lacking. Therefore, this study aims to compare the expression levels of PRGs between the normal bladder and bladder cancer, build a risk score for bladder cancer, determine its prognostic and clinical values, and explore its relationship with the immune microenvironment and immunotherapy biomarkers.

Material and Methods

Datasets

Data from RNA-sequencing (RNA-seq) and somatic mutation (VarScan2 Variant Aggregation and Masking), as well as the matched clinical characteristics used to construct the prognostic model, were downloaded from The Cancer Genome Atlas (TCGA) database (https://www.cancer.gov/about-nci/organization/ccg/research/structural-genomics/tcga). A total of 411 BC patient samples and 19 normal human bladder samples were obtained. RNA-seq and clinical data were downloaded from the GEO database (https://www.ncbi.nlm.nih. gov/geo/, ID: GSE13507, GSE31684) for external validation.

Identification of differentially expressed pyroptosis-related genes

Studies have already constructed a pyroptosis-related gene based prognostic model in other cancers, which were referred to in this study, after which a total of 33 pyroptosis-related genes were screened out[19-22], as shown in Table S1. For further comparison, the expression data of both datasets were normalized. The "limma" packages were then used to identify the differentially expressed genes (DEGs) between BC and normal tissues with a p-value <0.05, with the DEGs forming a heatmap. A protein-protein interaction (PPI) network was then constructed for 33 PRGs using the Search Tool for the Retrieval of Interacting Genes (STRING)，version 11.5 (https://string-db.org/). Interactionscore=0.9 was set as the lowest PPI network interaction score in order to find the hub genes.

Consensus Clustering

A clustering analysis was conducted for 411 bladder cancer patients using the "ConsensuClusterPlus" package, which was repeated 1000 times to make the stratification more stable. The number of clusters was determined by the K-means algorithm with the Euclidean distance. Patients were then divided into different pyroptosis modification patterns according to the K value.

Development and validation of a prognostic model based on pyroptosis-related gene

Univariate cox survival analysis was performed to evaluate the correlations between PRGs and prognosis in the TCGA cohort. Here, $p<0.05$ was set as the cut-off p-value, and the significant PRGs in the univariate Cox survival analysis were subsequently subjected to LASSO Cox regression analysis, with the minimum criteria deciding the penalty parameter (λ). The calculation formula of risk score was: Risk Score=Î£$_{i}^{5}$ Xi×Yi (X: coefficients, Y: gene expression level). BC patients in the TCGA and GEO groups were then divided into high and low-risk groups according to median risk score, and

Kaplan-Meier survival curves for OS were plotted using the survimer R package to compare the overall survival (OS) between the two groups. In order to ascertain the specificity and sensitivity of the risk score, time-dependent receiver operating characteristic (ROC) curves and AUC values were obtained using the survival ROC R package.

Identify independent prognostic factor for OS

The clinical data (age, sex, grade, T, N, and M) as well as the risk score of each patient in both the TCGA and GEO cohort were extracted. All indicators then underwent univariate Cox survival analysis, after which the statistically significant indicators ($p < 0.05$) were incorporated into the multivariate Cox survival analysis. These indicators in the multivariate Cox survival analysis ($p < 0.05$) were considered to be independent prognostic factors.

Develop a prognostic nomogram integrated clinical features and risk score

A nomogram was constructed based on the results of the multivariate Cox survival analysis. The prediction accuracy and discriminating ability of the nomogram were then evaluated using Harrell’s C-index and calibration curves, respectively. Furthermore, the time-dependent receiver operating characteristic (ROC) curve further evaluated predictive performance.

Functional enrichment analysis,tumor-infiltrating immune cells and immune-related pathways between the low and high-risk groups

Using the “limma” packages, $|log2FC|>log2(1.5)$ and FDR<0.05 were used as the screening standard for DEGs between the high and low-risk groups. According to these DEGs, the Gene Ontology (GO) enrichment and Kyoto Encyclopaedia of Genes and Genomes (KEGG) pathway analyses were performed by applying the "clusterProfiler" package. The single sample gene set enrichment analysis (ssGSEA) was then used to evaluate the enrichment scores of 16 tumor-infiltrating immune cells and 13 immune- related pathways in each sample from the TCGA group using the R package "gsva".

Tumor mutation burden and immune check point analysis

The somatic mutation data (VarScan2 Variant Aggregation and Masking) were obtained from The Cancer Genome Atlas (TCGA) database. Perl scripts based on JAVA8 was used to calculate the tumor mutational burden (TMB) of each patient, after which the median TMB was set as the cut-off value in order to divide the patients into high and low TMB groups. The Mann–Whitney test was used to compare the difference of tumor mutation burden (TMB) and immune check-point genes (PD1, PD-L1, CTLA4, LAG3 and TIM3) between the high and low-risk groups.

Results:

1. Identifying PRGs that are differentially expressed in bladder cancer and normal tissues

RNA sequencing (RNA-seq) data, along with the corresponding clinical data of 19 normal samples and 411 bladder cancer samples, were downloaded from the TCGA database. A heat map was constructed in order to show the differentially expressed PRGs between the normal and tumor samples (Figure 1A), of which a total of 15 PRGs were found to be differentially expressed in normal and tumor samples, while 11 genes (AIM2 GPX4 NLRP7, NLRP2, CASP3, CASP5, CASP6, CASP8, PYCARD PLCG1 GSDMD) were upregulated and 4 genes (IL6, NLRP3, ELANE, NLRP1) were downregulated in the BC samples. In order to explore the relationship among those PRGs, a Protein-Protein Interaction (PPI) network was implemented, with 0.9 set as the lowest PPI network interaction score. Here, 21 genes (CASP6, CASP9, CASP3, CASP8, CASP1, CASP4, CASP5, GZMB, TNF, IL6, IL1B, IL18, NOD1, NOD2, NLRP1, AIM2, GSDMD, NLRP6, NLRC4, NLRP3, PYCARD) were considered as hub genes (Figure 1B). Among them, 10 genes (AIM2, CASP3, CASP5, CASP6, CASP8, GSDMD, PLCG1, PYCARD, IL6, NLRP1) were both hub genes and differently expressed PRGs. The results of the correlation network with all PRGs are shown in Figure 1C.

2. Consensus Clustering of bladder cancer based on PRGs

In order to explore the relationship between PRGs and bladder cancer subtypes, an unsupervised consensus clustering analysis was conducted for 411 bladder cancer patients downloaded from the TCGA database. Accordingly, clustering variables k were found to increase from 2 to 10; when k = 2, the highest intragroup correlation and lowest intergroups correlation were obtained (Figure 2A). Therefore, patients were divided into two subtypes. The clinical features between the two subtypes are given in Figure 2B, where no significant differences were noted in regard to age, gender, T stage, N stage, M stage and grade between the two subtypes. Kaplan-Meier survival curves of OS were then performed in order to compare the difference in prognosis between the two subtypes. The 3-year survival rate of C1 and C2 was found to be 50.5% and 44.3%, respectively, while the 5-year survival rate of C1 and C2 was 43.1% and 34.8%, respectively. However, the survival curves showed no significant difference in OS between C1 and C2 (P =0.254). (Figure 2C)

3. Extracting prognosis associated PRGs and establishing risk scores in the TCGA datasets

In order to further assess the prognostic value of PRGs, PRG based risk scores were constructed to predict bladder cancer patient survival. Here, 403 patients with complete survival data in the TCGA data underwent univariate Cox survival analysis for 33 PRGs, of which 5 PRGs were found to be statistically significant (Figure 3A). LASSO regression analysis was then carried out in order to construct the prognostic model, with a penalty parameter (λ) of 5 (Figures 3B and 3C). The formula used to calculate risk score was: (-0.3168) * CASP6+ (-0.0849) * CASP8+(-0.0992) *AIM2+(-0.5139) * CASP9+(-0.1118) * GZMB. All patients were separated into the high-risk group (n=201, risk score≥-4.972744) and low-risk group (n=202, risk score<-4.972744) based on the median cut-off value. The Kaplan-Meier survival curve for OS showed that the high-risk group had a shorter survival time than the low-risk group ($p<0.001$) (Figure 3D). Moreover, the principal component analysis (PCA) showed that patients in different risk groups were distributed into two different groups (Figure 3E). Specifically, patients in the high-risk group had a higher possibility of death and a shorter survival time than those in the low-risk group (Figure 3F). A time-dependent ROC curve was then created in order to evaluate the performance of the prediction risk score. Accordingly, the AUC of the risk score in 1, 3 and 5 years was 0.667, 0.632 and 0.637, respectively (Figure 3G).

4. Validating the predictive efficiency of the risk model in the GEO cohort

In order to validate the replicability of the risk score in another patient group, PRG expression in 258 patients with complete survival data were obtained from the GEO cohort (GSE13507, GSE31684). The GEO dataset was then divided into the high-risk group (n= 94) and low-risk group (n=164) based on the median risk score cut-off. PCA analysis showed that patients in the two risk groups were also distributed according to two different groups (Figure 4A). As shown in Figure 4B, the high-risk group was noted to have more death events, while the low-risk group had a high probability of survival (Figure 4B). As illustrated by the Kaplan Meier curves for OS in Figure 4C, patients in the high-risk group were found to have a worse prognosis than those in the low-risk group ($P = 0.006$), with the high-risk group having a shorter survival time. To further assess the accuracy of the predictive risk model, a time-dependent ROC curve was analyzed. Here, the AUC of the model in 1, 3 and 5 years were found to be 0.621, 0.621 and 0.627, respectively (Figure 4D).

5. Risk score is an independent prognosis factor for OS

Patients were integrated from the TCGA and GEO datasets as a single dataset group so as to explore factors influencing prognosis. The clinical characteristics (age, sex, grade, T, N, and M) and

risk score in the two datasets were then analyzed via univariate and multivariate cox analyses. In the univariate cox analysis, variables including T, N, age, gender and risk score were found to serve as significant prognostic factors for prognosis（Figure 5A）. Moreover, risk score, gender, T, and N were verified as independent prognostic factors for OS according to the multivariate Cox regression analysis（Figure 5B）. In order to explore the relationship between risk score and clinical features, a heat map was constructed. As shown in Figure 5C, patients in the high-risk group were observed to be associated with advanced N and TNM stages.

6. A nomogram integrated risk scores and clinical signatures

To clarify the prognostic value of risk score in clinical application, a nomogram integrating clinical features and risk score was established. Gender, age, T, N and risk score were then verified as independent prognostic factors for OS in the multivariate Cox regression analysis and were used as variables to construct the nomogram (Figure 6A). The C-index for the nomogram was found to be 0.717 (CI 95%:0.635-0.799). The calibration curve demonstrated a high consistency between the actual survival rate and predicted survival rate in 3 and 5 years (Figures 6B and 6C). As seen in the ROC curve, the AUC of the nomogram in 3 and 5 years was found to be 0.789 and 0.775, respectively, which was higher than the AUC of gender, risk score and T+N stage (Figures 6D and 6E).

7. Functional analysis for PRG risk score

GO and KEGG pathway analyses were then conducted in order to explore the potential mechanism and biological function at the gene level for both the high and low-risk groups. The "limma" R package was employed to extract the differentially expressed genes (DEGs) between the two groups, with standards FDR<0.05 and |log2FC|>log2(1.5). A total of 122 DEGs were screened, of which 94 genes were found to be downregulated, while 28 genes were upregulated in the high-risk group. The GO analysis showed that the genes were more involved in immunity, especially in the biological process of antigen processing and presentation of exogenous peptide antigen, exogenous antigen, and peptide antigen (Figure 7A). Meanwhile, the KEGG analysis showed that these genes were also more correlated with antigen processing and presentation related pathways (Figure 7B).

8. The tumor-infiltrating immune cells and immune-related pathways between two risk groups.

In order to explore differences in the tumor microenvironment (TME) between the high and low-risk groups, a single-sample gene set enrichment analysis (ssGSEA) was conducted to evaluate the enrichment scores of 16 tumor-infiltrating immune cells and 13 immune-related pathways in each

sample from the TCGA and GEO group. The enrichment scores of the aDCs, CD8+_T_cells, pDCs, Tfh, Th2_cells, TIL and Treg in the low-risk group were found to be higher than those in the high-risk group in both the TCGA and GEO cohorts (Figures 8A and 8C). The enrichment level of DCs, NK_cells and Th1_cells were noted to be higher in the low-risk group in the TCGA cohort, while the enrichment level of B_cells, mast_cells, neutrophils and T_helper_cells were observed to be higher in the low-risk group in the GEO cohort. Figures 8B and 8D show that 7 immune-related pathways were found to be more significantly enriched in the low-risk group of both the TCGA and GEO cohorts, including APC_co_inhibition, Cytolytic_activity, HLA, Inflammation promoting, Parainflammation, T_CELL_co-stimulation and Type_I_IFN_Reponse. The immune-related pathway of checkpoint, MHC_class_I and T_cell_co-inhibition were shown to be higher in the low-risk group of the TCGA cohort, while APC_co_stimulation and CCR were higher in the low-risk group of the GEO cohort. These results suggest that the low-risk group had a higher abundance of tumor-infiltrating immune cells as well as a more active immune pathway compared to the high-risk group, which may explain the difference in prognosis between the two groups.

9. The difference in level of immunotherapy biomarkers between the two groups

In exploring the potential effect of immunotherapy in both groups, the difference of immunotherapy biomarkers was investigated between the two groups. Accordingly, heterogeneity in tumor mutation burden was noted between the two groups, with the low-risk group having a higher TMB compared to that of the high-risk group （Figure 9A）. The prognostic value of TMB in bladder cancer was also examined, in which the Kaplan-Meier survival curve showed that the high TMB group had a significantly longer OS than that of the low TMB group (p=0.005) (Figure 9B). In addition, the levels of other immunotherapy biomarkers were investigated (PD1, PD-L1, CTLA4, LAG3 and TIM3) in the high and low-risk groups. Here, PD1, PD-L1, CTLA4 and LAG3 were all noted to be highly expressed in the low-risk group, while TIM3 expression exhibited no difference between the two groups (Figure 9C-G). The corresponding findings suggest that patients in the low-risk group may potentially respond to immunotherapy.

Discussion

Pyroptosis is defined as inflammatory programmed death mediated by intracellular inflammasome

and the Gasdermin D (GSDMD) protein, which may have a dual effect on tumorigenesis and progression. Specifically, pyroptosis can significantly influence the immune-related signal cascade and further shape the inflammatory microenvironment, which may be beneficial to tumorigenesis; however, pyroptosis itself can lead to tumor cell death and carry out anti-tumor activities through modulation of the EMT process, regulation of the tumor microenvironment, and influencing chemotherapy resistance.[12-15] According to relevant studies, drugs inducing pyroptosis may have an impact on the synergism of radiotherapy and chemotherapy, reversal of chemotherapy resistance, regulation of body environment in shaping tumor metabolism, and enhancement response rate of the immune checkpoint inhibitor.[16-18] Moreover, tumor cells have been shown to hijack the caspase-9 pathway in order to inhibit radiotherapy, which may improve radiotherapy sensitivity.[35] In melanoma, low GSDME expression has been described to cause tumor cells to be resistant to etoposide, with the activation of pyroptosis reversing chemotherapy drug resistance. In addition, pyroptosis-inducing drugs may enhance the efficacy of immune checkpoint inhibitors (ICIs) and transform a "cold tumor" into a "hot tumor". ICIs have been widely used in the clinical management of malignant tumors; however, only one-third of patients respond to them [23]. The combination of ICIs and pyroptosis-inducing drugs may also make "cold tumors" respond [17]. One study found that inhibition of casp-9 can induce PD-L1 upregulation in colon cancer cells, demonstrating the potential efficacy of ICIs in conjunction with pyroptosis-inducing drugs.[16] Wang found that pyroptosis of less than 15% of tumor cells was sufficient to clear the entire 4T1 breast tumor xenograft in an animal model. Furthermore, in regard to “cold tumors” that do not respond to immune checkpoint inhibitors, the synergistic treatment of GSDM and PD1 inhibitors has been shown to trigger tumor response.[36]

This study constructed a pyroptosis gene signature prognostic model in bladder cancer. RNA-sequencing (RNA-seq) data, along with the matched clinical characteristics of bladder cancer patients, were obtained from the TCGA database, after which the risk score was established by conducting a univariate Cox survival and LASSO regression analysis. Eventually, 5 PRGs were screened so as to establish the prognostic model, with all being favorable factors for prognosis. According to the median risk score of the TCGA cohort, patients were divided into high and low-risk groups. In the TCGA cohort, the Kaplan-Meier survival curve showed that the OS of the high-risk group was significantly lower than that of the low-risk group. According to the ROC curve, the AUCs of the model at years 1, 3 and 5 were found to be 0.667, 0.632 and 0.637, respectively. In the GEO

cohort, the Kaplan-Meier survival curve also showed that the OS in the high-risk group was worse than that in the low-risk group. Meanwhile, the AUCs at years 1, 3 and 5 were 0.621, 0.621 and 0.627, respectively. A nomogram combined risk score and clinical features were then established in order to facilitate clinical use, of which the AUC of the nomogram in 3 and 5 years was found to be 0.789 and 0.775, respectively, higher than the AUC of gender, risk score and T+N stage. According to the GO and KEGG enrichment analyses, the DEGs between the two risk groups were shown to be more involved in antigen processing and presentation. In addition, a higher abundance of tumor-infiltrating immune cells and additional active immune pathways were observed in the low-risk group. Immunotherapy biomarkers were investigated in both groups, where the low-risk group was found to be rich in TMB, PD1, PD-L1, CTLA4, and LAG3. This suggests that patients in the low-risk group may potentially be responsive to immunotherapy.

Pyroptosis plays a bidirectional role in the occurrence and development of tumors. Activation of NLRP3 has been shown to promote the secretion of α-SMA and type I collagen as well as other fibrosis markers to promote liver fibrosis, thus promoting the occurrence of hepatocellular carcinoma. [37] However, in hepatocellular carcinoma tissues, the expression of NLPR3 has been shown to be negatively correlated with pathological grade and clinical stage [38], suggesting that apoptosis inhibits the further development of tumors. Similar results were evident in the present study, in which the genetic expression of 3 genes (AIM2, CASP6, and CASP8) were upregulated in tumor tissues and were validated as protective factors in the multivariate Cox regression analysis. Thus, these genes may play a similar role in bladder cancer as NLRP3 in liver cancer.

AIM2 is a pattern recognition receptor for the pyroptosis pathway, which is composed of the HIN-200 domain and N-terminal PYD. The HIN-200 domain binds with dsDNA, while the N-terminal PYD recruits ASC and CASP-1 to co-assemble into inflammasomes that dissociate the precursors of IL-1β and IL-18 into mature IL-1β and IL-18, thereby initiating cell pyroptosis [24]. In this study, high levels of AIM2 were found to be a protective factor for bladder cancer, though its level of expression and prognostic value were shown to be contradictory across different tumors. In colon cancer, decreased expression of AIM2 is known to be associated with advanced stages of cancer and tumor progression, while low AIM2 expression is considered to be indicative of poor prognosis.[25] In gastric cancer, the upregulation of AIM2 expression has been confirmed to promote tumorgenesis in mouse

models, while target blocking AIM2 can inhibit tumor occurrence. Moreover, the survival rate of patients with high AIM2 expression has been shown to be lower than those with low AIM2 expression[26]. Another study found that radiotherapy can activate the AIM2/ NLRP3-CasPA-IL-1 signaling pathway in mouse models, promoting tumor elimination.[27] Caspase-6 is considered to mediate the activation of apoptotic caspase [28], which is a crucial regulator of innate immunity, inflammasome activation, and host defense.[29] However, the function of Caspase-6 is not limited to apoptosis; it is a crucial component structure of ZBP1-Panoptosome, which can induce PANoptosis (' P', Pyroptosis; 'A', Apoptosis; 'N', Necroptosis; and 'optosis') to prevent IAV infection.[30] Few studies pertaining to caspase-6 in malignant tumors exist, however. This study showed that caspase-6 serves as a prognostic factor for bladder cancer, though further investigation is needed to explore the role of caspase-6 in the pyroptosis-related pathway as well as ascertain its clinical value. Caspase- 8 is the molecular switch of apoptosis, necrosis and pyroptosis, [31] and activated caspase-8 can directly cleave GSDMD, thereby inducing cell pyroptosis.[32, 33] Caspase-8 expression levels were found to be lower in normal brain tissue compared to those in malignant glioma, while patients with overexpression of caspase-8 were in the earlier stages. At the cellular level, caspase-8 can inhibit malignant glioma cell proliferation.[34] Studies have shown that tumor cells suppress radiation-induced immunity by hijacking caspase-9 signaling. Moreover, inhibiting caspase-9 can induce tumor cells to produce type I interferon, increase activity of tumor-specific CD8+T cells, delay tumor cell growth, and increase radiotherapy sensitivity.[16] The findings of the present study suggest that targeted CASP-9 inhibitors may have the clinical potential to enhance the efficacy of radiotherapy. In triple-negative breast cancer, Mir-224 has been shown to downregulate caspase-9 expression and promote tumor growth.[35] CZMB acts as a molecular weapon for cytotoxic lymphocytes in defending against viral infection and malignant transformation. [39] GZMB rapidly cleaves CASP-3 in target cells and activates the CASP-3/ GSDME-mediated pyroptosis pathway [40]. A recent study has also shown that GZMB can cleave GSDME and directly induce cell pyroptosis [41]. Furthermore, in patients with stage IV non-small cell lung cancer who were treated with ICIs, high levels of GZMB resulted in better OS and PFS.[42] In colon tumors, GZMB has been shown to be highly expressed in the proximal colon and is characterized by MSI-high and BRAF mutations, with higher levels of GZMB being associated with longer OS and CSS.[43] The aforementioned studies all indicate the potential of utilizing pyroptosis genes in clinical settings.

Pyroptosis-related target drugs possess great potential in the development of tumor drugs. Induced pyroptosis can activate anti-tumor immunity, suggesting that the combination of immune chec kpoint inhibitors and pyroptosis inducing drugs may lead to a higher response rate. Inhibition to cell apoptosis serves as the mechanism of tumor resistance to chemotherapy and radiotherapy. Cell pyroptosis is a new form of programmed cell death, and the development of drugs targeting pyroptosis may serve as a novel approach in overcoming drug resistance. The heterogeneity and deletion of tumor antigens are one of the reasons for the failure of adoptive immunotherapy. [44] The pyroptosis of tumor cells can induce the release of tumor antigens, promote the maturation of DCB cells, initiate T cell cloning and proliferation, and activate anti-tumor immunity, which may be beneficial in order to enhance the efficacy of adoptive immunotherapy.

This study has several limitations. First, the number of normal tissue samples in the TCGA cohort was relatively small, which may lead to bias. Second, only one case of non-muscle invasive bladder cancer was present in the data; hence, the results may be limited to muscle-invasive bladder cancer. In addition, in vivo and in vitro experiments are necessary to support the findings of this study.

Author Contributions

Xiangyu Zhang and Fangzhou Guo contributed the central idea, analysed most of the data, and wrote the initial draft of the paper. The remaining authors contributed to refining the ideas, carrying out additional analyses and finalizing this paper.

Conflict of Interest Statement

The authors declare that the research was conducted in the absence of any commercial or financial relationships that could be construed as a potential conflict of interest.

Statement

Bladder cancer is the most common malignant tumor in the urinary system with a poor prognosis.Pyrotopia, as a novel form of programmed cell death, has great potential for tumor treatment. Our study construct a risk score composed of pyroptosis-related genes, which can predict the prognosis

of bladder cancer. We found that risk scores were associated with immune cell infiltration, immune-related pathways, and immunotherapy biomarker.

Reference

1,Richters A, Aben KKH, Kiemeney LALM, et al.The global burden of urinary bladder cancer:an update.World J Urol. 2020;38(8):1895-1904.

2,Ferlay J et al (2018) Global cancer observatory: cancer today. Available from:https://gco.iarc.fr/today. Accessed date 01 May 2019.

3,Torre LA, Bray F, Siegel RL, et al. Global cancer statistics, 2012.CA Cancer J Clin.2015;65:87–108.

4,Kirkali Z,Chan T,Manoharan M,et al.Bladder cancer: epidemiology,staging and grading,and diagnosis.Urology. 2005;66(6)(suppl 1):4-34.

5,Ghandour R,Singla N,Lotan Y,et al.Treatment Options and Outcomes in Nonmetastatic Muscle Invasive Bladder Cancer.Trends Cancer,2019 Jul;5(7):426-439.

6,G Facchini,C Cavaliere,L Romis,et al.Advanced/metastatic bladder cancer: current status and future directions.Eur Rev Med Pharmacol Sci,2020 Nov;24(22):11536-11552.

7,Dyrskjøt L,Ingersoll MA.Biology of nonmuscle-invasive bladder cancer:

pathology,genomic implications,and immunology[J]. Curr Opin Urol,2018,28(6):598-603.

8,Lenis AT,Lec PM,Chamie K,et al.Bladder Cancer: A Review.JAMA.2020 Nov 17;324(19):1980-1991.

9,Bergsbaken T,Fink SL,Cookson BT,et al.Pyroptosis: host cell death and inflammation[J].Nat Rev Microbiol,2009,7(2):99-109.

10,Jorgensen I,Rayamajhi M, Miao E A. Programmed cell death as a defence against infection.Nat Rev Immunol,2017,17:151–164.

11,Aachoui Y,Leaf IA,Hagar JA,et al.Caspase-11 protects against bacteria that escape the vacuole.Science,2013,339: 975–978.

12,Chen YT,Su LP,Qiu XY,et al.Molecular mechanism of pyroptosis and its role in cancer (in Chinese). Sci Sin Vitae, 2020,50:1042–1054.

13,Dupaul-Chicoine J, Arabzadeh A, Dagenais M, et al. The Nlrp3 inflammasome suppresses colorectal cancer metastatic growth in the liver by promoting natural killer cell tumoricidal activity[J].Immunity,2015,43(4):751-763.

14,Wei Q,Mu K,Li T,et al.Deregulation of the NLRP3 inflammasome in hepatic parenchymal cells during liver cancer progression[J].Lab Invest,2014,94(1):52-62.

15,Lu H,Zhang S,Wu J,et al.Molecular targeted therapies elicit concurrent apoptotic and GSDME- dependent pyroptotic tumor cell death[J].Clin Cancer Res,2018,24(23):6066-6077.

16,Han C, Zhang Y, Liu Z.Tumor cells suppress radiation-induced immunity by hijacking caspase 9 signaling.Nature Immunology.2020,6;21(5):546-554.

17,Tang R,Xu J,Zhang B.Ferroptosis,necroptosis,and pyroptosis in anticancer immunity.J Hematol Oncol,2020 Aug 10;13(1):110.

18,Zhu xy,LJ.New Strategies of Inhibiting Malignant Tumor Based on Inducing Pyroptosis.Cancer Research on Prevention and Treatment.2021;48(4);393-399.

19,Ye Y,Dai Q,Qi H.A novel defined pyroptosis-related gene signature for predicting the prognosis of ovarian cancer.Cell Death Discov.2021;7:71.

20,Lin WL,Chen Y,Wu BM,et al.Identification of the pyroptosis related prognostic gene signature and the associated regulation axis in lung adenocarcinoma.Cell Death Discov,2021 Jun 25;7(1):161.

21,Li XY,Zhang LY,Li XY,et al.A Pyroptosis-Related Gene Signature for Predicting Survival in Glioblastoma.Front Oncol

,2021 Aug 17;11:697198.

22,Shao W,Yang ZC,Fu Y,et al.The Pyroptosis-Related Signature Predicts Prognosis and Indicates Immune Microenvironment Infiltration in Gastric Cancer.Front Cell Dev Biol,2021 Jun 11;9:676485.

23,Jiang P,Gu S,Pan D,et al.Signatures of T cell dysfunction and exclusion predict cancer immunotherapy response. Nat Med. 2018;24:1550–8.

24,Yu P ,Zhang X,Liu N,et al.Pyroptosis: mechanisms and diseases.Signal Transduct Target Ther,2021 Mar 29;6(1):128.

25,Zhao J,LI J,Shi G.Expression of AIM2 and serum CEA level in patients with colorectal cancer and its clinical significance. Chinese Journal of Colorectal Diseases.2019,8(3):257-261.

26,Dawson RE,Deswaerte V,West AC.STAT3-mediated upregulation of the AIM2 DNA sensor links innate immunity with cell migration to promote epithelial tumourigenesis.Gut,2021 Sep 6;gut jnl-2020-323916.

27,Han C,Victoria G,Liu Z.The AIM2 and NLRP3 inflammasomes trigger IL-1–mediated antitumor effects during radiation.Sci Immunol,2021 May 7;6(59):eabc6998.

28,Opdenbosch NV, Lamkanfi M. Caspases in Cell Death, Inflammation, and Disease. Immunity. 2019 6 18;50(6):1352–64.

29,Foveau B,Kraak LVD,Beauchemin N,et al. Inflammation- induced tumorigenesis in mouse colon is caspase-6 independent. PLoS One,2014 Dec 3;9(12):e114270.

30,Zheng M,Kanneganti TD.Newly Identified Function of Caspase-6 in ZBP1-mediated Innate Immune Responses,NLRP3 Inflammasome Activation, PANoptosis, and Host Defense .J Cell Immunol.2020;2(6):341–347.

31,Fritsch M,Günther SD, Schwarzer R,et al. Caspase- 8 is the molecular switch for apoptosis,necroptosis and pyroptosis.Nature,2019,Nov;575(7784):683-687.

32,Orning P,Weng D, Starheim Ket al.Pathogen blockade of TAK1 triggers caspase-8–dependent cleavage of gasdermin D and cell death.Science.2018 Nov 30;362(6418):1064-1069.

33,Sarhana J, Liu BC,Muendlein HI,et al. Muendleinc.Caspase-8 induces cleavage of gasdermin D to elicit pyroptosis during Yersinia infection.Proc Natl Acad Sci U S A,2018 Nov 13;115(46):E10888-E10897.

34,Wang HB,Li T,Ma DZ,et al.Overexpression of FADD and Caspase-8 inhibits proliferation and promotes apoptosis of human glioblastoma cells.Biomed Pharmacother,2017 Sep;93:1-7.

35,Zhang L,Zhang X,Wang X,et al.MicroRNA-224 Promotes Tumorigenesis through Downregulation of Caspase-9 in Triple-Negative Breast Cancer.Dis Markers,2019 Feb 11;2019:7378967.

36.Wang, Q., Wang, Y., Ding, J. et al. A bioorthogonal system reveals antitumour immune function of pyroptosis. Nature 579, 421–426 (2020).

Li J, Zhao YR, Tian Z. Roles of hepatic stellate cells in acute liver failure: From the perspective of inflammation and fibrosis. World J Hepatol 2019; 11(5): 412-420.

38.Wei, Q., Mu, K., Li, T. et al. Deregulation of the NLRP3 inflammasome in hepatic parenchymal cells during liver cancer progression. Lab Invest 94, 52–62 (2014).

39,Kurschus FC,Jenne DE.Delivery and therapeutic potential of human granzyme B.Immunol Rev,2010 May;235(1):159-71.

40,Liu YY,Fang YL,Chen XF,et al.Gasdermin E-mediated target cell pyroptosis by CAR T cells triggers cytokine release syndrome.Sci.Immunol.5,eaax7969 (2020).

41,Zhang ZB,Zhang Y,Xia SY,et al.Gasdermin E suppresses tumour growth by activating anti-tumour immunity. Nature 579, 415–420 (2020).

42,Hurkmans DP,Basak EA,Schepers N,et al.Granzyme B is correlated with clinical outcome after PD-1 blockade in patients with stage IV non-small-cell lung cancer.J Immunother Cancer,2020 May;8(1):e000586.

43,Prizment AE,Vierkant RA,Smyrk TC.Cytotoxic T Cells and Granzyme B Associated with Improved Colorectal Cancer Survival in a Prospective Cohort of Older Women.Cancer Epidemiol Biomarkers Prev,2017 Apr;26(4):622-631.

44.Titov A, Zmievskaya E, Ganeeva I, ,et al. Adoptive Immunotherapy beyond CAR T-Cells. Cancers (Basel). 2021 Feb 11;13(4):743.

Figure legends

Figure 1A. Heat map shows the expression levels of 15 differentially expressed PRGs between normal and tumor samples.

Figure 1B. A protein-protein interaction (PPI) network for PRGs was constructed using the Search Tool for the Retrieval of intervening Genes (STRING). PPI network shows the interaction of 21 hub genes in PRGs.

Figure 1C. The correlation network contains all 33 PRGs.

Figure 2A. Consensus score matrix of bladder cancer samples when K = 2.

Figure 2B. The heat map shows the clinical characteristics between the two subtypes, including age, sex, grade,T , N , M , stages.

Figure 2C..Kaplan-Meier survival curves for OS in C1 and C2 subtypes.

Figure 3A. The Forest plot shows that 5 PRGs were potential prognostic factors for OS in univariate cox survival analysis.

Figure 3B. LASSO regression analysis of the 5 PRGs.

Figure 3C. LASSO regression analysis of the 5 PRGs.

Figure 3D..Kaplan Meier curves for OS in bladder cancer patients (n=403) who had been stratified into high and low-risk groups based on the median risk score.

Figure 3E. PCA plot shows the distribution of high-risk and low-risk groups.

Figure 3F. The distribution of survival status for the high-risk and low-risk groups.

Figure 3G. ROC curves of 1,3 and 5 years for the risk score.

Figure 4A. PCA plot shows the distribution of high-risk and low-risk groups.

Figure 4B. The distribution of survival status for the high-risk and low-risk groups.

Figure 4C. Kaplan Meier curves of OS for the high-risk and low-risk groups (P =0.006).

Figure 4D. ROC curves of 1,3 and 5 years for the risk score.

Figure 5A. Univariate cox regression analysis of OS for clinical characteristics and risk score.

Figure 5B. Multivariate Cox regression analysis of clinical characteristics and risk score.

Figure 5C. Heatmap showed the connections between clinical characteristics and risk score.

Figure 6A. Nomograms intergrated clinical characteristics and risk score for predicting OS based on all the patients in two cohort .

Figure 6B. The calibration curves predicting 3 year OS.

Figure 6C. The calibration curves predicting 5 year OS.

Figure 6D. ROC curve in 3 year to assess the AUC of the gender,nomogram, T+Nstage.

Figure 6E. ROC curve in 5 year to assess the AUC of the gender,nomogram, T+Nstage.

Figure 7A. The bubble graph showed the analysis of GO enrichment for DEGs between two risk groups.

Figure 7B. The Barplot graph showed the analysis of KEGG enrichment for DEGs between two risk groups.

Figure 8A. Enrichment levels of 16 tumor-infiltrating immune cells in high and low-risk groups from TCGA data.

Figure 8B. Enrichment levels of 13 immune-related pathways in high and low-risk groups from TCGA data.

Figure 8C. Enrichment levels of 16 tumor-infiltrating immune cells in high and low-risk groups from GEO data.

Figure 8D. Enrichment levels of 13 immune-related pathways in high and low-risk groups from GEO data.

Figure 9A.The level of TMB in the high and low- risk groups

Figure 9B. Kaplan Meiercurvesof OS for the high and low-TMB groups(P=0.005)

Fig9C-G. The level of immune checkpoints（PD1, PD-L1,TIM3, LAG3, CTLA4 ） in the high and low- risk groups

Figure 1

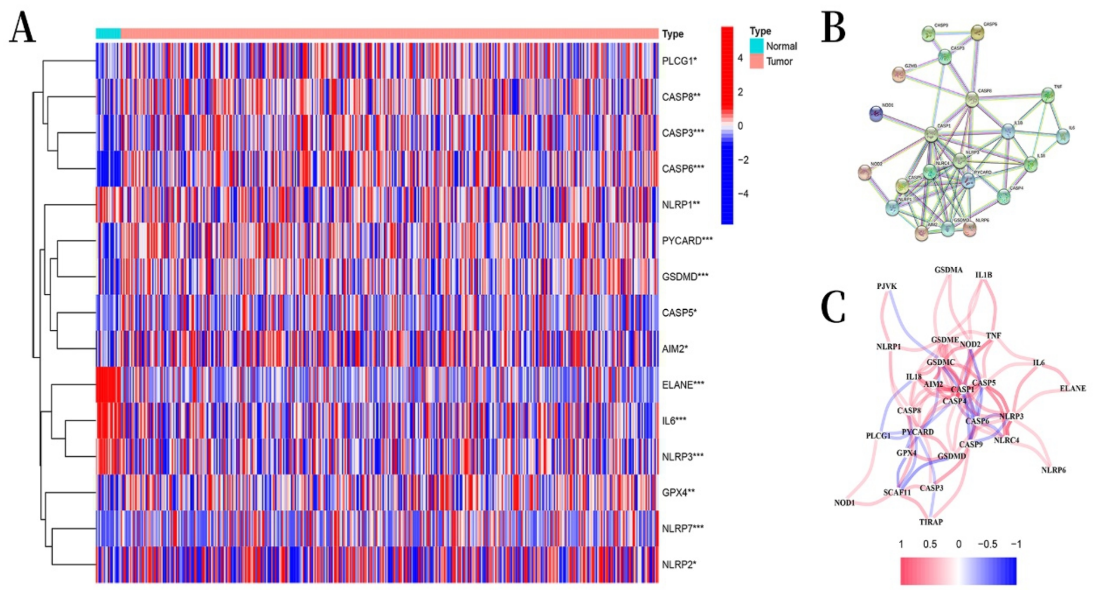

Figure 2

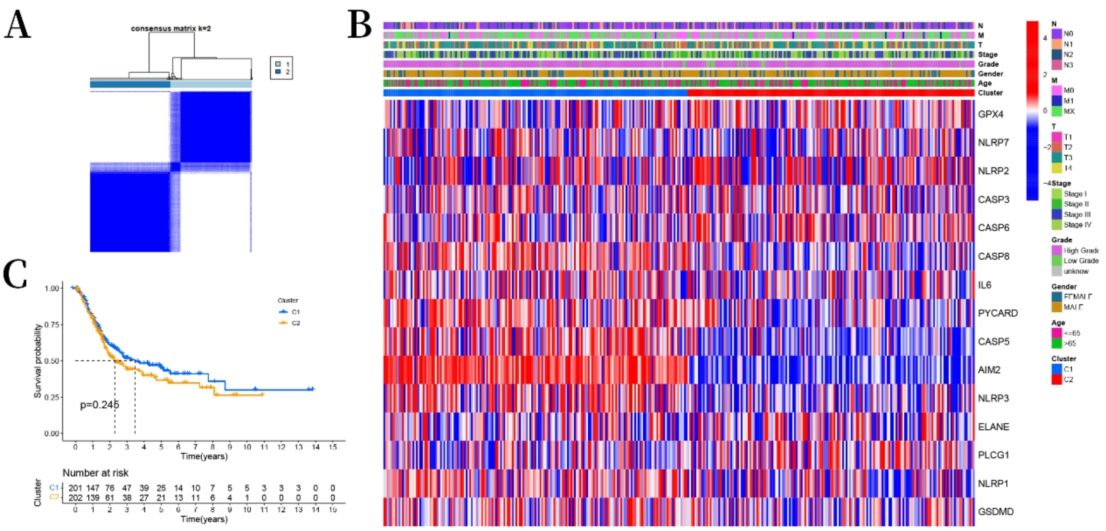

Figure 3

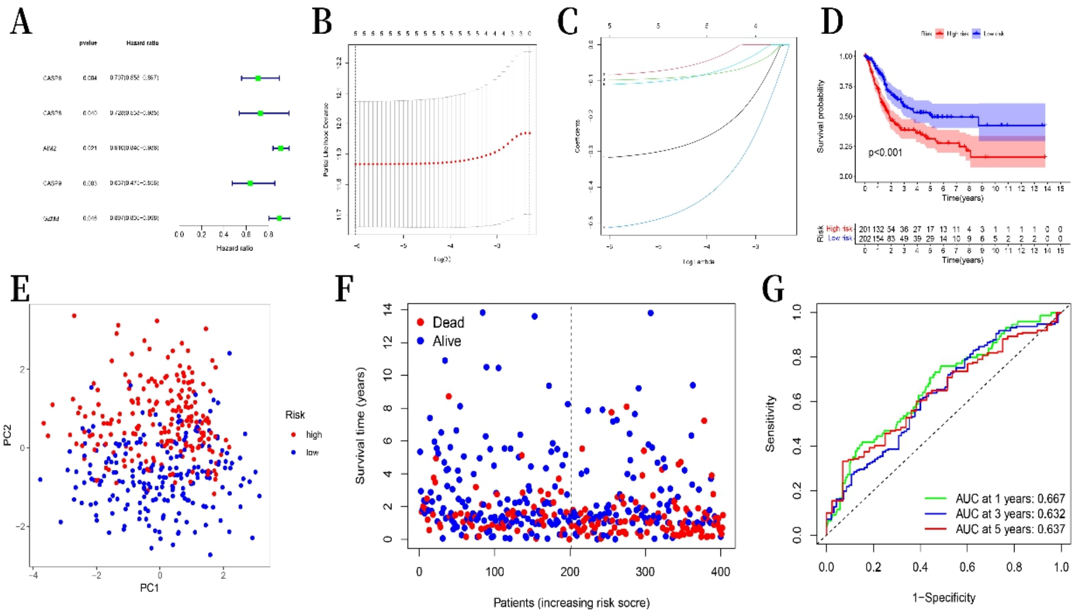

Figure 4

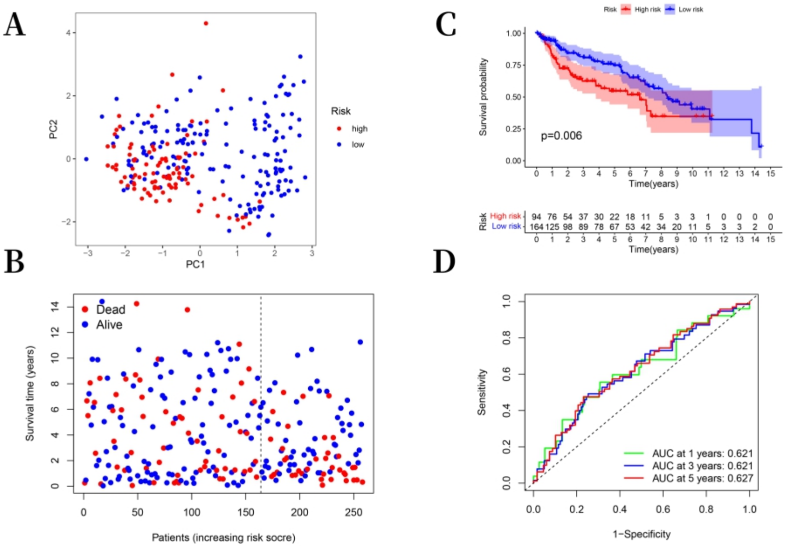

Figure 5

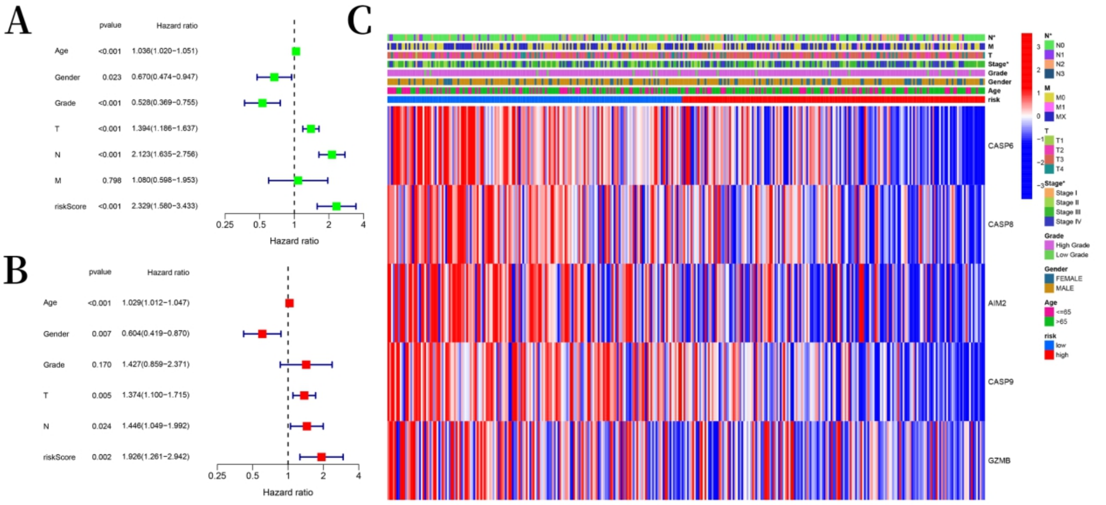

Figure 6

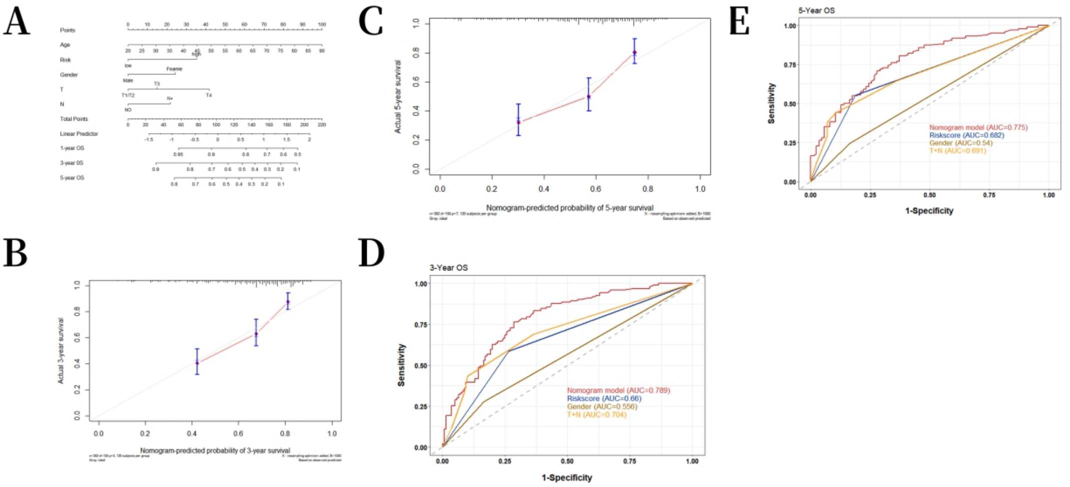

Figure 7

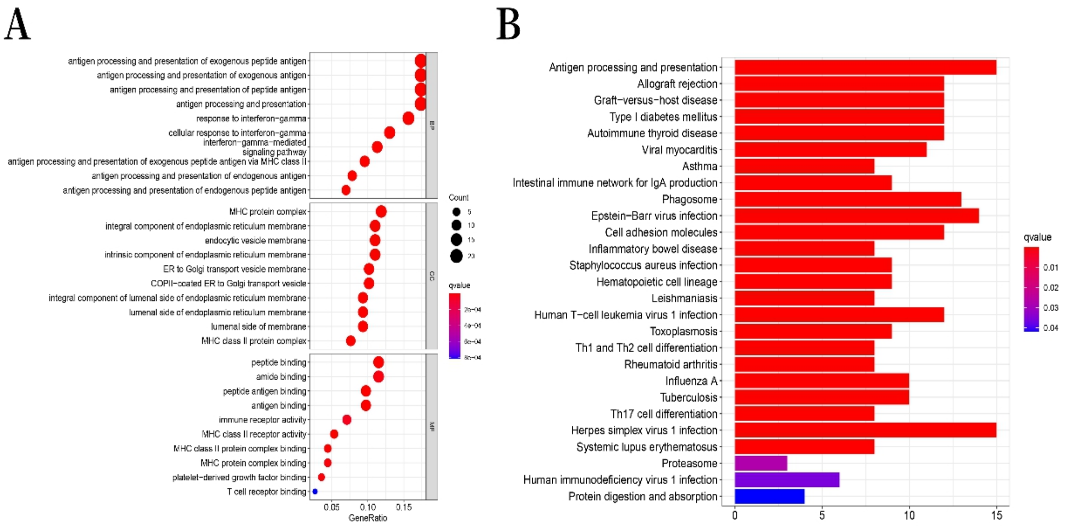
A
antigen processing and presentation of exogenous peptide antigen
antigen processing and presentation of exogenous antigen
antigen processing and presentation of peptide antigen
antigen processing and presentation
response to interferon−gamma
cellular response to interferon−gamma
interferon−gamma−mediated signaling pathway
antigen processing and presentation of exogenous peptide antigen via MHC class II
antigen processing and presentation of endogenous antigen
antigen processing and presentation of endogenous peptide antigen
MHC protein complex
integral component of endoplasmic reticulum membrane
endocytic vesicle membrane
intrinsic component of endoplasmic reticulum membrane
ER to Golgi transport vesicle membrane
COPII−coated ER to Golgi transport vesicle
integral component of lumenal side of endoplasmic reticulum membrane
lumenal side of endoplasmic reticulum membrane
lumenal side of membrane
MHC class II protein complex
peptide binding
amide binding
peptide antigen binding
antigen binding
immune receptor activity
MHC class II receptor activity
MHC class II protein complex binding
MHC protein complex binding
platelet−derived growth factor binding
T cell receptor binding
BP
CC
MF
0.05
0.10
0.15
GeneRatio
Count
5
10
15
20
qvalue
B
Antigen processing and presentation
Allograft rejection
Graft−versus−host disease
Type I diabetes mellitus
Autoimmune thyroid disease
Viral myocarditis
Asthma
Intestinal immune network for IgA production
Phagosome
Epstein−Barr virus infection
Cell adhesion molecules
Inflammatory bowel disease
Staphylococcus aureus infection
Hematopoietic cell lineage
Leishmaniasis
Human T−cell leukemia virus 1 infection
Toxoplasmosis
Th1 and Th2 cell differentiation
Rheumatoid arthritis
Influenza A
Tuberculosis
Th17 cell differentiation
Herpes simplex virus 1 infection
Systemic lupus erythematosus
Proteasome
Human immunodeficiency virus 1 infection
Protein digestion and absorption
0
5
10
15
qvalue
0.01
0.02
0.03
0.04

Figure 8

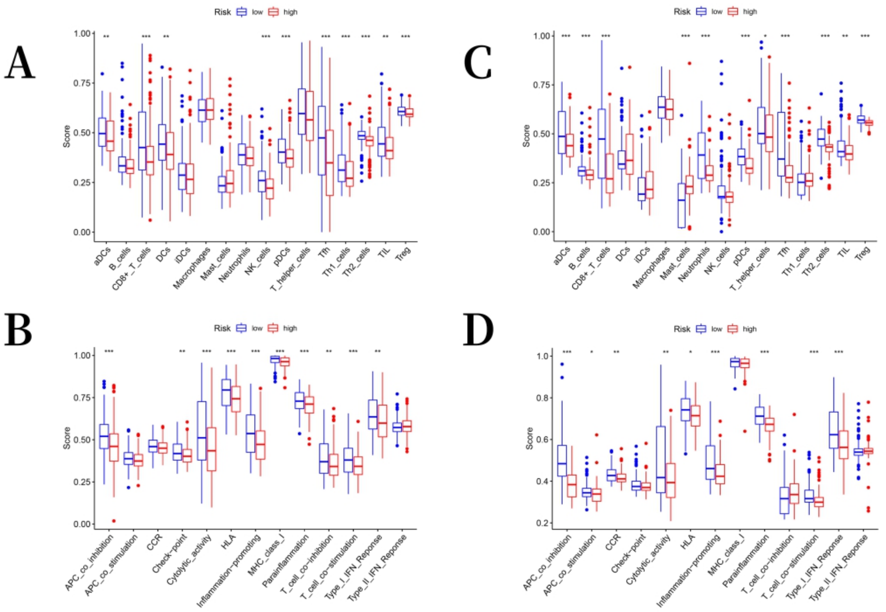
A
B
C
D
Risk low high
Score

Figure 9

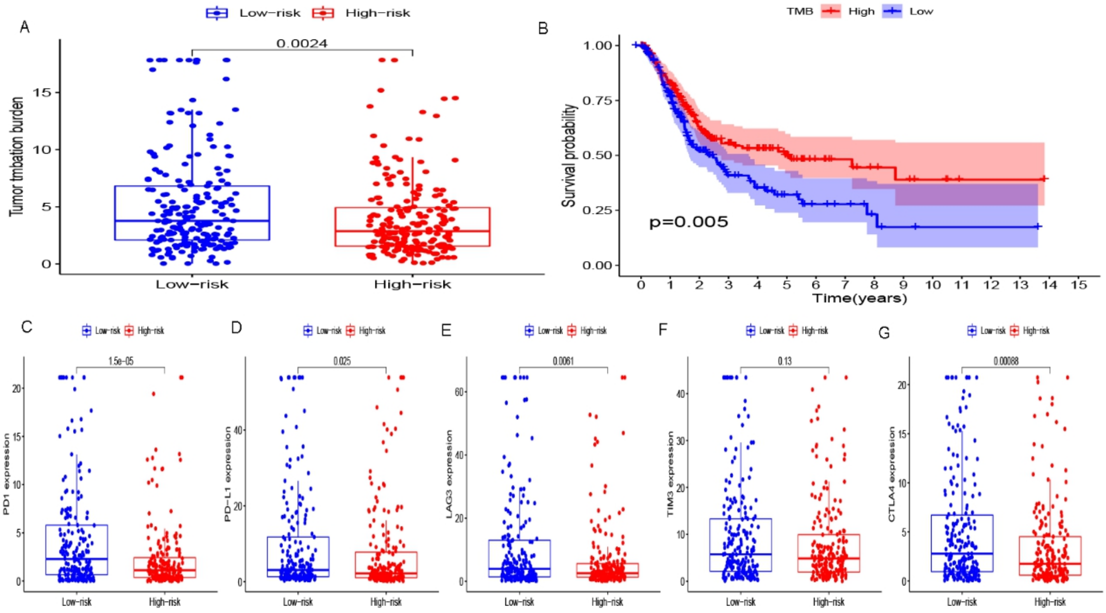
A
Low-risk High-risk
0.0024
Tumor tmbation burden
Low-risk
High-risk
B
TMB High Low
Survival probability
p=0.005
Time(years)
C
1.5e-05
PD1 expression
D
0.025
PD-L1 expression
E
0.0061
LAG3 expression
F
0.13
TIM3 expression
G
0.00088
CTLA4 expression
